# Lymph Node-Targeted Synthetically Glycosylated Antigen Leads to Antigen-Specific Immunological Tolerance

**DOI:** 10.3389/fimmu.2021.714842

**Published:** 2021-09-24

**Authors:** Chitavi D. Maulloo, Shijie Cao, Elyse A. Watkins, Michal M. Raczy, Ani. S. Solanki, Mindy Nguyen, Joseph W. Reda, Ha-Na Shim, D. Scott Wilson, Melody A. Swartz, Jeffrey A. Hubbell

**Affiliations:** ^1^ Pritzker School of Molecular Engineering, University of Chicago, Chicago, IL, United States; ^2^ Animal Resources Center, University of Chicago, Chicago, IL, United States; ^3^ Biomedical Engineering Department, Johns Hopkins University, Baltimore, MD, United States; ^4^ Committee on Immunology, University of Chicago, Chicago, IL, United States; ^5^ Ben May Department of Cancer Research, University of Chicago, Chicago, IL, United States; ^6^ Committee on Cancer Biology, University of Chicago, Chicago, IL, United States

**Keywords:** lymph node, subcutaneous, tolerance, glycopolymer, regulatory T cell, dendritic cell, lymphatics, co-inhibition

## Abstract

Inverse vaccines that tolerogenically target antigens to antigen-presenting cells (APCs) offer promise in prevention of immunity to allergens and protein drugs and treatment of autoimmunity. We have previously shown that targeting hepatic APCs through intravenous injection of synthetically glycosylated antigen leads to effective induction of antigen-specific immunological tolerance. Here, we demonstrate that targeting these glycoconjugates to lymph node (LN) APCs under homeostatic conditions leads to local and increased accumulation in the LNs compared to unmodified antigen and induces a tolerogenic state both locally and systemically. Subcutaneous administration directs the polymeric glycoconjugate to the draining LN, where the glycoconjugated antigen generates robust antigen-specific CD4^+^ and CD8^+^ T cell tolerance and hypo-responsiveness to antigenic challenge *via* a number of mechanisms, including clonal deletion, anergy of activated T cells, and expansion of regulatory T cells. Lag-3 up-regulation on CD4^+^ and CD8^+^ T cells represents an essential mechanism of suppression. Additionally, presentation of antigen released from the glycoconjugate to naïve T cells is mediated mainly by LN-resident CD8^+^ and CD11b^+^ dendritic cells. Thus, here we demonstrate that antigen targeting *via* synthetic glycosylation to impart affinity for APC scavenger receptors generates tolerance when LN dendritic cells are the cellular target.

## Introduction

Current treatments for autoimmune and inflammatory diseases are non-curative and rely on broad nonspecific immunosuppression, risking a number off-target effects, complications, and opportunistic infections that limit the long-term use of these strategies. As the underlying mechanisms of immune suppression and the identities of the disease-causing autoantigens and allergens are being increasingly unraveled, antigen-specific therapies are being put through the clinical developmental pipeline to a greater extent (1, 2). Several strategies to induce a more directed antigen-specific immune response are under investigation (3). Subcutaneously-administered free antigens have been explored, including in clinical trials, in the context of celiac disease, diabetes and multiple sclerosis (4–6). Delivery and formulation approaches have also been explored to direct antigen to APCs for preferential uptake without activation and subsequent tolerogenic education of naïve T cells (7–11). Successful strategies have included coating PLGA microparticles with DEC-205^+^ DC-targeting antibodies or P-D2 integrin-targeting peptides (8) or encapsulating PLGA nanoparticles with antigen (such as MOG peptide) and immunosuppressive agents such as IL-10 (9) or rapamycin (10) to promote tolerogenic DC maturation. However, these approaches are still limited in efficacy due to immunogenicity of the vehicle or ADAs that can result from repeated dosing.

Antigen glycosylation has been leveraged as an immune-modulatory tool in the context of both vaccination and tolerance (12). Since glycan binding to carbohydrate-binding receptors is a low-affinity event, multivalency of glycosylation has been shown to be beneficial in the optimal engagement of these receptors (13). Covalent attachment of carbohydrate structures from pathogens or cancer cells to immunogenic proteins has been explored to improve the efficacy of activating or tolerogenic vaccines (14). Moreover, antigens modified with glycosylation repeats have been used to target a number of lectin receptors such as the asialoglycoprotein receptor (15), DC-SIGN (16), MARCO receptor (17), and LSECtin (18).

We have shown in prior work that antigens decorated with synthetic glycopolymers of N-acetyl glucosamine (p(GluNAc)) or N-acetyl galactosamine, after intravenous (i.v.) injection, promiscuously target various subsets of hepatic APCs, resulting in antigen-specific tolerance (19, 20). Here, we investigate this approach to target draining lymph node (dLN)-resident APCs, seeking to understand whether tolerance can be induced *via* peripheral subcutaneous (s.c.) administration and to elucidate the mechanisms involved. We show that antigen-p(GluNAc) is retained to a higher extent in the dLNs, improving uptake by APCs and promoting antigen presentation so as to generate a pool of long-lived anergic antigen-specific CD4^+^ and CD8^+^ T cells in addition to regulatory T (Treg) cells that attenuate effector T cell responses and maintain tolerance in the face of an inflammatory antigenic challenge. We also explore differences in immunological mechanisms between tolerization *via* the LN, accessed *via* s.c. administration, and liver, *via* i.v. administration, with synthetically glycosylated antigen. Thus, we present a subcutaneously-administered biocompatible inverse vaccine platform that is promising for blunting the response to antigens, such as primary autoantigens, allergens, or protein drugs, opening the approach of glycoconjugate inverse vaccination to a new APC subset with a convenient route of administration.

## Materials and Methods

### Study Design

The objective of this study was to target synthetically glycosylated antigen to LN APCs to induce antigen-specific immunological tolerance, and investigate the molecular mechanisms of tolerance. We delivered p(GluNAc)-conjugated antigen to dLNs *via* s.c. administration, and characterized the antigen distribution, retention and uptake landscape, as well as downstream effects on the antigen-specific T cell response. We furthermore elucidated the contribution of specific APC subsets, T cell regulatory populations, and co-stimulatory signaling axes to the maintenance of tolerance. Flow cytometry and fluorescence microscopy were the primary analytical techniques used, and the OTI and OTII TCR-transgenic system was the main model studied. The number of experimental replicated are indicated in figure legends.

### Mice

Mice were maintained in a pathogen-free facility at the University of Chicago. All experiments and procedures in this study were performed with the approval of the Institutional Animal Care and Use Committee at the University of Chicago. Female C57BL/6N mice, aged 7-12 weeks, were purchased from Charles Rivers (strain code: 027). OTI (JAX code: 003831) and OTII (JAX code: 004194) were crossed to CD45.1^+^ mice (JAX code: 002014) to yield congenically labeled OTI and OTII mice. Batf3^-/-^ mice (also on a C57BL/6 background) were originally a donation from Justin P. Kline’s laboratory at the University of Chicago, and subsequently, bred in house.

### OVA-p(GluNAc) Synthesis and Characterization

Detailed synthesis and characterization methods can be found in (19). Briefly, p(GluNAc) was synthesized using a reversible addition-fragmentation chain transfer (RAFT) polymerization using an azide-modified RAFT agent, a biologically inert comonomer (N-(2-hydroxypropyl) methacrylamide, HPMA) and the glycosylated methacrylamide N-acetyl glucosamine monomer. We use a copper-free click-based reaction in aqueous solvent at room temperature to conjugate the polymers to antigens to preserve the antigen’s tertiary structure and function. To this end, the OVA (Invivogen, vac-pova) is modified at terminal amines with an amine-reactive heterobifunctional bicyclononyne-decorated linker. Upon conjugation, this linker forms a reduction-sensitive chemical bond that is stable in serum but is cleaved when the conjugate encounters the reductive environment of the endosome inside the antigen presenting cell. The polymer ranges in size from 30-60 kDa, and can be visualized on a non-reducing SDS-page gel after conjugation to antigen. Conjugated OVA-p(GluNAc) was separated from unconjugated OVA by size exclusion in PBS buffer and the concentration of conjugated OVA was quantified by boiling the conjugate in reducing Laemmli buffer and running it on a reducing SDS-page gel alongside unmodified OVA samples of known concentrations. Finally, OVA-p(GluNAc) was tested for the presence of endotoxin before being used in tolerance experiments. For the synthesis of the fluorescent OVA_647_-p(GluNAc) conjugate, Alexa Fluor™ 647 NHS ester (Thermo Fisher Scientific A20006) was first conjugated to OVA before the click linker step.

### S.c. Tolerization

Unless otherwise specified, mice were injected s.c. in all four hocks at a dose of 5 μg of OVA antigen and volume of 20 μL per hock, under isoflurane anesthesia.

### Whole-Organ Fluorescence Imaging of LNs

15 h after s.c. hock injections, whole cardiac perfusion was performed with PBS (pH= 7.4) under isoflurane inhalation anesthesia, after which the liver and draining axillary and popliteal LNs were isolated. The organs were cleaned by removing extra fatty tissue and washed in PBS to remove blood that could contribute to auto-fluorescence. They were imaged on the *In Vivo* Imaging System (IVIS, PerkinElmer) using an excitation wavelength of 630 nm and an emission wavelength of 650 nm. For the time-dependent antigen retention study, mice were sacrificed without perfusion at timepoints of 1 h, 6 h, 15 h, 24 h, 48 h and 72 h post-injection and draining popliteal LNs were isolated and imaged using the same procedure described above.

### LN APC Biodistribution

24 h after s.c. hock injection, mice were sacrificed and draining LNs were isolated. The LN capsule was gently poked with 25 G needles. They were digested at 37°C, first with 1 mg/mL Collagenase IV and 40 μg/mL DNAse1 for 30 min, followed by 3.3 mg/mL Collagenase D and 40 μg/mL DNAse1 in 300 μL of DMEM (Gibco 11966025) supplemented with 1.2 mM CaCl_2_ for 15 min with magnetic stirring. The LNs were gently pipetted 100 times using an electronic pipette. An equal volume of ice-cold 10 mM EDTA in PBS supplemented with 1% FBS was added to the digestion mixes to quench the enzymatic reaction for a final concentration of 5 mM EDTA, followed by pipetting for another 100 times. The cell suspensions were filtered through a 70 μM filter to generate a single cell suspension which was stained for flow cytometry. Antibodies against the following markers were used: CD45 – APC-Cy7 (BioLegend, 103116), CD31 – BV421 (BioLegend, 102423), GP38 – PE-Cy7 (eBioscience 25-5381-82), CD21/35 – FITC (BioLegend, 123407), B220 – BUV496 (BD Biosciences 564662), CD3e – BUV395 (BD Biosciences 563565), CD11c – PE (BioLegend 117308), CD11b – BV785 (BioLegend 101243), CD8 – BUV737 (BD Biosciences 612759), CD103 – PE (BD Biosciences, 561043), CD169 – BV421/BV605 (BioLegend 142421/142413), MerTK – PerCP-eF710 (eBioscience 46-5751-82), CX3CR1 – PE (BioLegend 149005), F4/80 – BUV395 (BD Biosciences 565614), and MHCII – FITC/PerCP-Cy5.5 (BioLegend 107605/BD Biosciences 612759).

### Whole Mount Confocal Imaging of LN

Popliteal lymph nodes were fixed in Zinc (pH= 6.5) at 4°C for 24 h. The LNs were washed with tris buffered saline (TBS) and permeabilized with filtered tris buffered saline (TBS) 1% Triton X-100 5% DMSO (pH= 7.4) for 12 h at RT to degrade intracellular fat that could interfere with the staining. The LNs were washed and gently digested with a mixture of Collagenase IV (1 mg/mL), DNAse1 (40 μg/ml) and Collagenase D (3.3 mg/mL) enzymes in 0.5% casein in TBS supplemented with 5 mM CaCl_2_ for 45 min at room temperature. LNs were incubated with unlabeled or biotinylated primary antibodies at 1 μg/mL in 0.5% casein in TBS overnight at 4°C. 10 μg of DNAse1 was added to the primary antibody mix as an added precaution. After thoroughly washing with 0.1% Tween TBS, followed by TBS, the LNs were gently dried and stained with secondary labeled or streptavidin conjugated F(ab)_2_ at 3.75 μg/mL in 0.5% casein in TBS overnight at 4°C. The LNs were thoroughly washed, dried, and dehydrated by sequentially washing in 70%, 95% and finally 100% ethanol. LNs were gently compressed on a microscopy slide, mounted with 25 mg/mL of propylgalate in a 2:1 solution of benzyl benzoate in benzyl alcohol (BABB). The cover slip was placed on the LN and edges were sealed using silicone glue. The mounted LNs were imaged using an Olympus confocal microscope equipped with CellSense software. Images were acquired using four lasers (488 nm, 594 nm, 647 nm and 750 nm excitation wavelengths) and a confocal stack, and analyzed using Imaris 9.1.2 software.

### Adoptive Transfer of OTI CD8^+^ and OTII CD4^+^ T Cells

CD8^+^ T cells were isolated from the spleen and s.c. LNs (axillary, brachial, inguinal, popliteal, cervical) of OTI mice using the EasySep CD8^+^ isolation kit (Stemcell 19853). Similarly, CD4^+^ T cells were isolated from the spleen and s.c. LNs of OTII mice using the EasySep CD4^+^ isolation kit (Stemcell 19852). Spleens were first mashed into a single cell suspension and lysed with ACK lysis buffer (Gibco A1049201). LNs were digested with 1 mg/mL Ca^2+^ supplemented Collagenase D (Roche 11088866001) for 45 min at 37°C and gently mashed into a single cell suspension. Suspensions from the LNs and spleen were pooled and subjected to magnetic cell isolation using the kits. The OTI and OTII cells were labeled with 1 μM CFSE (eBioScience 65-0850-84) for 6 min at RT, washed with sterile PBS buffer, quantified and resuspended in saline buffer for injection. 5x10^5^ - 1x10^6^ cells of each OTI and OTII cells were injected into mice *via* i.v. tail vein injection.

### Challenge Following Adoptive Transfer and Tolerization

Mice received an inflammatory s.c. challenge of 20 ug EndoFit OVA (InvivoGen vac-pova) and 50 ng LPS (Sigma) total in all four hocks under isoflurane anesthesia. Mice were sacrificed under CO_2_ inhalation 5 days following challenge.

### Preparation of Cell Suspensions for Flow Cytometry Analysis

Draining s.c. LNs (axillary and popliteal) and the spleen were isolated from mice. Spleens were first mashed into a single cell suspension with plain DMEM media (Gibco 11966025), filtered through 70 μM cell strainers, and lysed with ACK lysis buffer (Gibco A1049201). LNs were digested with 1 mg/mL Ca^2+^ supplemented Collagenase D (Roche 11088866001) for 45 min at 37°C and gently mashed into a single cell suspension, also with DMEM and through 70 μM cell strainers. The cells were resuspended in IMDM media (Gibco 12440053), supplemented with 10% FBS and 1% Penicillin-Streptomycin (Gibco 15140122), and counted using a LUNA automated fluorescent cell counter (Logos biosystems). Cells were seeded at a count of 1x10^6^ - 3x10^6^ per well in 96-well round-bottom plates for subsequent antibody staining for flow cytometry. Antibodies against the following markers were used: CD3 – BUV395 (BD Biosciences 563565), CD8-BUV737 (BD Biosciences 612759), CD4 – BUV496 (BD Biosciences 612952), Foxp3 – FITC (BD Biosciences 560403), CD25 – BV605 (BioLegend 120235), ST2 – BV421 (BD Biosciences 566309), Lag3 – PerCP-Cy5.5 (BD Biosciences 564673), CTLA4 – PE-Cy7 (eBioScience 17-1522-82), IFNγ – APC (BioLegend 505810), TNFα – BV605 (BioLegend 506329), IL-2 – FITC (BioLegend 503806), IL-10 – APC-Cy7 (BioLegend 505036), PD-1 – BV711 (BioLegend 135231), Tim3 – PE (BD Biosciences 566346).

### *Ex Vivo* Antigen-Specific Restimulation

LN and spleen single-cell suspensions were seeded at a count of 1x10^6^ - 3x10^6^ per well in non-tissue culture treated round-bottom 96-well plates (Celltreat 229590), and stimulated *ex vivo* at 37°C for 2 h with either OVA_257-264_ peptide (Genscript) at a final concentration of 1 μg/mL, or OVA_323-339_ peptide (Genscript) at 2 μg/mL, followed by Brefeldin A at a final concentration of 5 μg/mL for another 4 h. The cells were then washed with PBS before proceeding with cytokine antibody staining for flow cytometry. For long-term restimulations, grade V OVA (Sigma A5503) was added to cells at a final concentration of 100 μg/mL for 4 days. The culture supernatant was collected and frozen for subsequent cytokine ELISA (ThermoFisher Scientific 88-7314-77) and LegendPlex™ (BioLegend 741044) assays.

### *In Vivo* Blockade of Co-Stimulatory Molecules

Mice were administered *via* i.p. injection 250 μg of either αLag-3 (BioXCell BE0174, clone C9B7W), αPD-1 (BioXCell BE0146, clone RMP1-14) or αCTLA-4 (BioXCell BE0164, clone 9D9) on days 1, 3, 5, 7, 9 and 11 for a total of 6 injections.

### *In Vivo* Macrophage Depletion

For the depletion study in **Figure S5A**, mice were treated s.c. in all four hocks with 250 μg of αCSF1R or an isotype IgG2a control once only on day 0 and sacrificed on day 7 to evaluate macrophage depletion. For the experiment described in **Figure 5A**, mice were treated s.c. in all four hocks with 250 μg of αCSF1R (BioXCell BE0213, clone AFS98) or an isotype IgG2a control (BioXCell BE0089, clone 2A3) on days 0, 3, 6 and 9.

### *Ex Vivo* DC Sorting and Priming

Pooled s.c. LNs (axillary, brachial, inguinal, popliteal, cervical) were isolated from wild-type mice, and digested into a single-cell suspension as described in the “LN APC biodistribution” section above. All reagents were kept sterile and all procedures were handled in a biosafety hood when possible. The cell suspension was washed with MACS buffer and the following biotinylated antibodies were added at a final concentration of 2 μg/mL to deplete specific cell populations: αCD3 (T cells), αCD19 (B cells), αB220 (B cells), αGr-1 (neutrophils), and αNK1.1 (NK cells). The cells were washed with MACS buffer, resuspended with Dynabeads Biotin Binder (Invitrogen 11047), and placed in a magnet. The depleted LN suspension was carefully pipetted out of the tube and washed with MACS buffer before proceeding with antibody staining for FACS. The following antibodies were added at these specified dilutions in FACS buffer: Streptavidin – APC-Cy7 (1:400, BD Biosciences 47-4317-82), CD64 – PE-Cy7 (1:100, BioLegend 139314), F4/80 – PE-Cy7 (1:100, BioLegend, 123114), CD11c – APC (1:200, BioLegend 117310), MHCII – PacBlue (1:800, BioLegend, 107620), CD8α – PerCP-Cy5.5 (1:200, BioLegend, 100734), CD103 – PE (1:100, BD Biosciences, 561043), and CD11b – BV510 (1:400, BioLegend 101263). The cells were washed before staining with near-IR Live-Dead dye in PBS, and resuspended in MACS buffer for sorting. The cells were then sorted into four populations: CD8^+^ resident (CD11c^+^MHCII^int^CD8^+^CD11b^-^, denoted as CD8^+^ rDC1), CD103^+^ migratory (CD11c^+^MHCII^high^CD103^+^CD11b^-^, denoted as CD103^+^ mDC1), CD11b^+^ resident (CD11c^+^ MHCII^int^CD8^-^CD11b^+^, denoted as CD11b^+^ rDC2) and CD11b^+^ migratory (CD11c^+^MHCII^high^CD103^-^CD11b^+^, denoted as CD11b^+^ mDC2) (see **Figure S5D** for the gating strategy). The sorted cells were collected in sterile RPMI media supplemented with 10% FBS, 1% Penicillin-Streptomycin, 0.1% Gentamicin and 50 μM β-mercaptoethanol. The DC populations were counted and plated at a number of 2.7 × 10^4^ per well in triplicates in a 96-well round bottom plate. Each population was then stimulated in a 1:1 ratio with CFSE-labeled OTI and OTII cells in the presence of 2 μM of unmodified OVA or OVA-p(GluNAc) at 37 ^0^C. Three days later, the cells were harvested, and the OTI and OTII cells were analyzed for proliferation and activation (CD44^+^).

### *Ex Vivo* Culture of Primary LN-LECs and Priming

LNs (axillary, brachial, popliteal, inguinal, cervical) were isolated from female WT C57BL/6 mice into plain RPMI medium. They were gently poked with 29G1/2 needles and transferred into digestion media made up of 0.25 mg/mL Liberase DH and 200 Kunitz/mL DNAse1 in RPMI media for a total of 1 h at 37°C. Every 10-15 min, the LNs were poked and the digest was pipetted up and down. At the end of 45 min, a single cells suspension is obtained, and filtered through a 70 μM cell strainer into a 50 mL conical. This was spun down, resuspended in 10 mL of αMEM media containing 1% P/S and 10% FBS and seeded into a T75 tissue culture flask at 37°C. The T75 flask was coated with a mixture of 10 μg/mL collagen I and 10 μg/mL human plasma fibronectin in 1x PBS for 30 min at 37°C prior to transferring the cells. The cells were washed with 1x PBS 24 h and 72 h post-isolation and 10 mL of fresh complete αMEM media was replaced. At day 5 post-isolation, the adhered lymph node stromal cells (~85% LECs) were detached from the surface of the T75 flask by first washing with 1x PBS and adding accutase for ~7 min at RT. The detached cells were transferred into a 50 mL conical, spun down and resuspended in complete αMEM media. These were counted and plated at a density of 2.7 × 10^4^ per well in a flat-bottom 96-well plate. The LECs were stimulated in a 1:1 ratio with CFSE-labeled OTI and OTII cells in the presence of 2 μM of unmodified OVA or OVA-p(GluNAc) at 37 ^0^C. Three days later, the cells were harvested, and the OTI and OTII cells were analyzed for proliferation and activation (CD44^+^).

### Statistical Analysis

Statistically significant differences between experimental groups were determined using Prism software (version 6.07, GraphPad). All n values and statistical analyses are stated specifically in the figure legends for all experiments. For most experiments, a one-way or two-way ANOVA, followed by Tukey’s or Dunnett’s post-hoc test was used. Comparisons were significant if p < 0.05. For two-group comparisons, unpaired Student’s T test was used.

## Results

**Figure 1:**
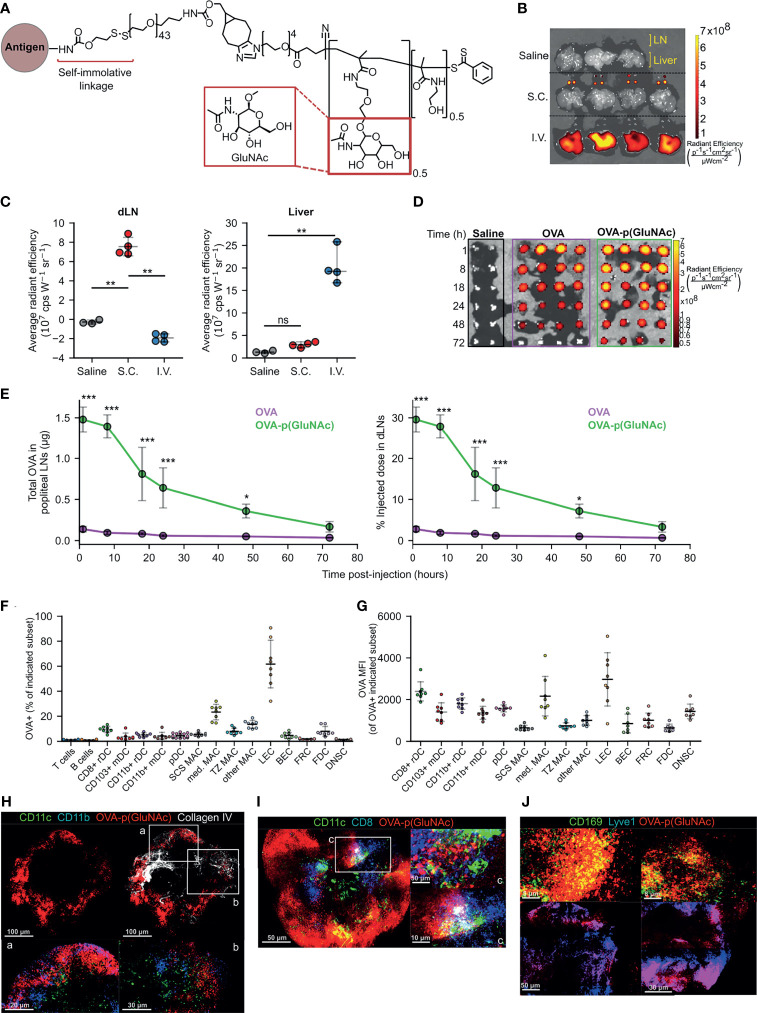
**(A)** Structure of p(GluNAc) conjugated to an antigen lysyl side chain amine *via* a self-immolative linker. **(B, C)** Accumulation of OVA_647_ or OVA_647_-p(GluNAc) in the dLNs and livers 15 h after s.c. or i.v. injection. **(B)** Representative NIR images of dLNs and liver. **(C)** Average NIR intensities of dLNs and livers. **(D, E)** Mice were injected with saline or 5 μg of OVA_647_ or OVA_647_-p(GluNAc) s.c. and the draining popliteal LNs were isolated and imaged at various timepoints between 1-72 h post-injection n (dLN)= 2 for saline and 4 for OVA_647_ or OVA_647_-p(GluNAc) at each timepoint. **(D)** Representative NIR images of dLNs. **(E)** Quantification of OVA accumulation in dLNs, expressed as μg (left) or % of initial injected dose (right). **(F, G)** Flow cytometry analysis of LN cells that took up OVA_647_-p(GluNAc) 15 h after s.c. injection of 20 μg of OVA_647_-p(GluNAc). DC, dendritic cell; pDC, plasmacytoid dendritic cell; MAC, macrophage; SCS, subcapsular sinus; med, medullary; TZ MAC, T cell zone macrophage; LEC, lymphatic endothelial cell (CD45^-^CD31^+^gp38^+^); BEC, blood endothelial cell (CD45^-^CD31^+^gp38^-^); FRC, fibroblastic reticular cell (CD45^-^CD31^-^gp38^+^CD21/35^-^); FDC, follicular dendritic cell (CD45^-^CD31^-^gp38^+^CD21/35^+^); DNSC, double negative stromal cell (CD45^-^CD31^-^gp38^-^). **(F)** Percent of each cell subset that is OVA^+^. **(G)** OVA_647_ MFI of each OVA^+^ cell subset. **(H–J)** Representative whole mount confocal images of immunostained dLNs after s.c. injection of OVA_647_-p(GluNAc) (red). **(H)** LNs stained versus CD11c (green), CD11b (blue) and Collagen IV (white), 8 h post-injection (p.i.). CD11c^+^CD11b^+^ DCs are shown in aqua and co-localized OVA-p(GluNAc) is shown in magenta. Scale bar ranges from 20-100 μm and is indicated in each panel. **(I)** LNs stained versus CD11c (green) and CD8 (blue) 18 h p.i. CD11c^+^CD8^+^ double positive DCs are shown in aqua and co-localized OVA-p(GluNAc) is shown in magenta. Scale bar ranges from 5-50 μm and is indicated in each panel. **(J)** LNs stained versus CD169 (green, top) and Lyve1 (blue, bottom). Co-localized OVA-p(GluNAc) with CD169^+^ macrophages is shown in yellow (top), and OVA-p(GluNAc) co-localized with the lymphatics is shown in magenta (bottom). Scale bar ranges from 5-50 μm and is indicated in each panel. Data represent mean ± SD. *p ≤ 0.05, **p ≤ 0.01, ***p ≤ 0.001, ns is not significant by one-way ANOVA using Tukey’s *post hoc* test in **(C)**, and two-way ANOVA using Sidak’s *post hoc* test in **(E)**.

Our previous work describes the synthesis of antigen-glycopolymer conjugates composed of synthetic polymers synthesized from N-acetylglucosamine-decorated monomers conjugated to protein or peptide antigens *via* a self-immolative linker that cleaves in response to intracellular stimuli (19) (**Figure 1A**). When injected i.v., our antigen-N-acetylglucosamine glycopolymer (p(GluNAc)) conjugates accumulate in the liver and are taken up by hepatic APCs. Upon delivery to hepatic APCs, our self-immolative linker is cleaved from the antigen, which releases the conjugated antigen in its unmodified form to allow efficient antigen processing and presentation by hepatic APCs (19). Here, we seek to understand the nature and extent to which LN APC populations can induce antigen T cell non-responsiveness and regulation when collecting the glyco-antigen under homeostatic conditions.

### Antigen-p(GluNAc) Conjugate Injected Subcutaneously Accumulates in the Draining Lymph Nodes Where it Targets Various Subsets of Antigen Presenting Cells

We first determined whether s.c. injected glyco-polymerized antigen, in this case ovalbumin (OVA)-p(GluNAc), is specifically retained in the draining LNs (dLN), which we expected due to its optimal size and molecular weight (~100 kDa) for lymphatic uptake (21). Indeed, we were able to detect OVA-p(GluNAc) in the draining axillary and popliteal LNs (dLNs), using whole-organ fluorescence imaging, only when injected s.c. in the hocks but not after an i.v. injection (**Figures 1B, C**). Conversely, OVA-p(GluNAc) was only detected in the liver when injected i.v. but not s.c. (**Figures 1B, C**). We also verified that no antigen remained at the site of immunization 72 h after injection (**Figure S1A**). This demonstrates the versatility and unique trafficking profile of our synthetically glycosylated antigen platform depending on the injection route.

Following s.c. injection of an equivalent antigen dose and visualization of the fluorescence in the dLNs at different timepoints after injection, we found that OVA-p(GluNAc) localizes to the dLNs to a higher extent than unconjugated OVA (**Figure 1D**). The higher accumulation of OVA-p(GluNAc) is expressed in two ways: as absolute protein content calculated from a dose-radiant efficiency standard curve, and as a percent of the initially injected antigen dose per hock of 5 μg (**Figure 1E**). We detected a 17-fold difference in antigen accumulated (maximum at time = 8 h), and a 10-fold difference in the area under the curve, in the favor of OVA-p(GluNAc) (**Figure 1E**). Increased antigen retention in the first few days of immunization is especially important under unadjuvanted conditions where a higher antigen dose and availability need to trump transient TCR-pMHC interactions for fruitful T cell stimulation to occur (22, 23).

After confirming that antigen-p(GluNAc) accumulated in the dLN, we verified whether antigen was taken up by APCs in the LN microenvironment. We conducted a biodistribution experiment in which we assessed the types of APCs that took up antigen-p(GluNAc) and the extent to which they did 15 h after s.c. injection. OVA-p(GluNAc) was taken up by different APC types, reported as % OVA^+^ within each APC subset (**Figure 1F**) or mean fluorescence intensity (MFI) of OVA^+^ cells **(** **Figure 1G**). These APCs included various subsets of macrophages, dendritic cells and lymphatic endothelial cells (LECs) that efficiently take up antigen due to their strategic location within the LN, their phagocytic ability and expression of scavenger receptors (24–28). Using multi-parameter flow cytometry, we elucidated the contribution of specific APC subsets that took up antigen after administration of OVA-p(GluNAc). Among these were APCs found at and surveilling the subcapsular sinus such as LECs (26), CD169^+^ subcapsular sinus macrophages (27) and CD11b^+^ resident DC2s (24) as well as APCs that are more deeply located in the medullary or cortical regions of the LN, such as the CD169^+^ medullary macrophages (29), cross-presenting resident CD8^+^ DC1s (30) and T cell zone CX3CR1^+^Mertk^+^ macrophages (28). These results confirm that antigen-p(GluNAc) can traffic and be taken up by APCs located at different locations within the LN for subsequent processing and presentation (**Figures 1F, G**).

To obtain a visual confirmation for our flow cytometry results, we isolated popliteal LNs from mice that had been injected s.c. in the hind hocks with fluorescently-labeled OVA_647_-p(GluNAc) and imaged whole mounts on a confocal microscope. We stained APCs using a combination of CD11c and CD11b for non-cross presenting DC2s (**Figure 1H**), or CD11c and CD8 for resident cross-presenting DCs (**Figure 1I**), or CD169 for subcapsular sinus and medullary macrophages **(** **Figure 1J**). We also stained for the basement membrane and lymphatics using antibodies to collagen IV and Lyve1, respectively (**Figures 1H, J**). OVA-p(GluNAc) was found to promiscuously co-localize with all the APC subsets imaged and mentioned above, consistent with our flow cytometry results and indicating that the mechanism of action is not preferential targeting of specific APC subsets but increased antigen uptake by LN APCs in general.

### Antigen-p(GluNAc) Leads to Antigen-Specific CD4^+^ and CD8^+^ T Cell Tolerance, Induction of Regulatory Subsets and Hypo-Responsiveness Upon Antigenic Challenge

**Figure 2:**
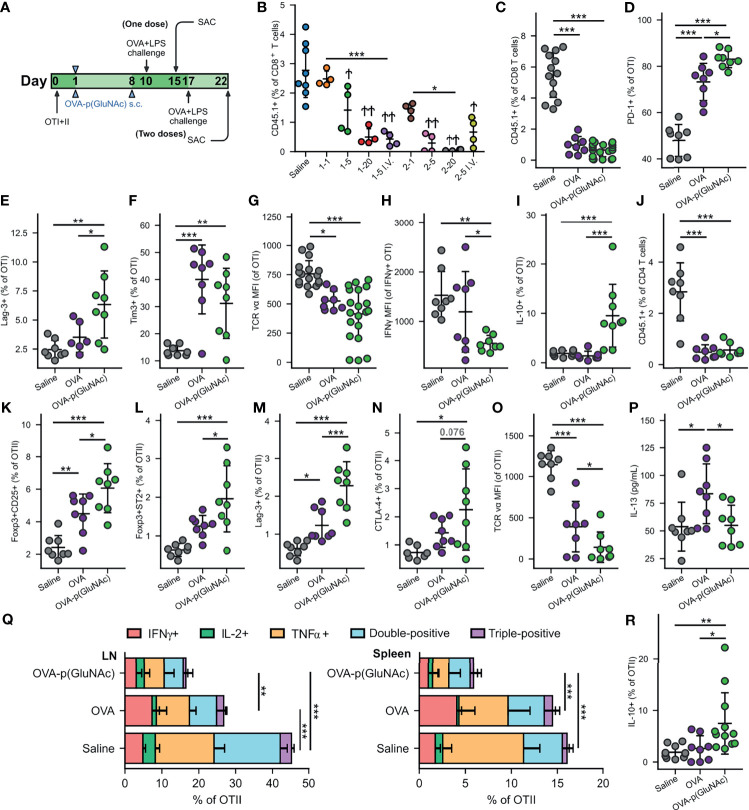
**(A)** Experimental timeline of the dose-efficacy study, n=4. CD45.2^+^ mice that had received an adoptive transfer of both OTI (CD45.1^+^CD3^+^CD8^+^) and OTII (CD45.1^+^CD3^+^CD4^+^) T cells *via* i.v. injection, were treated with saline or a low, mid or high dose of OVA-p(GluNAc) s.c. in all four hocks or i.v. in the tail vein (as benchmark) either once on day 1 or twice on days 1 and 8. 9 days following the last dose, on day 10 (for the groups that received one dose) or on day 17 (for the groups that received two doses), all mice were administered an OVA+LPS challenge s.c., and 5 days later, the dLNs and spleen were examined for an OVA-specific response. Stars above horizontal bars represent p values with respect to the i.v. groups (*p ≤ 0.05, **p ≤ 0.01, ***p ≤ 0.001) and † indicate p values with respect to the saline group (^†^p ≤ 0.05, ^††^p ≤ 0.01). **(B)** OTI CD8^+^ T cells recovered from dLNs at time of sacrifice. Plot legends are as follows: 1-1 (1 μg s.c., once), 15 (5 μg s.c., once), 1-20 (20 μg s.c., once), 1-5 i.v. (5 μg i.v., once), 2-1 (1 μg s.c., twice), 2-5 (5 μg s.c., twice), 2-20 (20 μg s.c., twice) and 2-5 i.v. (5 μg i.v., twice). **(C–R)** Data are representative of three pooled experiments performed at the optimal high 20 μg dose of OVA as unconjugated OVA or OVA-p(GluNAc) injected twice on days 1 and 8, followed by an OVA+LPS challenge on day 17 and sacrifice on day 22, n=8-20. **(C)** OTI CD8^+^ T cells recovered from dLNs. **(D)** PD-1^+^ OTI CD8^+^ T cells in dLNs. **(E)** Lag-3^+^ OTI CD8^+^ T cells in spleen. **(F)** Tim-3^+^ OTI CD8^+^ T cells in spleen. **(G)** MFI of the TCR on OTI CD8^+^ T cells in dLNs. **(H)** IFNγ MFI of IFNγ secreting OTI cells after a 6-h *ex vivo* restimulation with OVA_257-264_ peptide. **(I)** IL-10 producing OTI CD8^+^ T cells after a 6-h *ex vivo* restimulation with OVA_257-264_ peptide. **(K)** OTII CD4^+^ T cells recovered from dLNs. **(L)** Foxp3^+^CD25^+^ OTII CD4^+^ Tregs induced in dLNs. **(M)** Foxp3^+^ST2^+^ OTII CD4^+^ Tregs induced in dLNs. **(N)** Lag3^+^ OTII CD4^+^ T cells in dLNs. **(O)** CTLA-4^+^ OTII CD4^+^ T cells in dLNs. **(P)** MFI of the TCR on OTII CD4^+^ T cells in dLNs. **(P)** IL-13 levels in the supernatant of LN cells restimulated with 100 μg/mL OVA protein for 4 days, measured by LegendPlex assay. **(Q)** OTII CD4^+^ T cells from the dLNs (left) or spleen (right) that secreted IFNγ, IL-2, TNFα, or a combination of two or all three cytokines after a 6-h *ex vivo* restimulation with OVA_323-339_ peptide. **(R)** IL-10 producing OTII CD4^+^ T cells after a 6-h *ex vivo* restimulation with OVA_323-339_ peptide. Data represent mean ± SD. *p ≤ 0.05, **p ≤ 0.01, ***p ≤ 0.001 by one-way ANOVA using Tukey’s *post hoc* test.

We used the OTI/II OVA-reactive transgenic T cell receptor (TCR) model to assess the impact of s.c. administration of OVA-p(GluNAc) on the immune response. We adoptively transferred naïve CD45.1^+^ OTI CD8^+^ and OTII CD4^+^ T cells into mice one day before they were injected s.c. with OVA-p(GluNAc) or saline as unimmunized control, and compared that to i.v. administered OVA-p(GluNAc), as we have published (19). We first determined the optimal dose at which OVA-p(GluNAc) was tolerogenic through the s.c. route. We also compared the effect of immunizing mice one vs. two times with the same molecule. We challenged mice with OVA and LPS 9 days following injection (for mice that received one dose) or 9 days following the second dose (for mice that received two doses) and assessed the OVA-specific immune response 5 days after challenge (**Figure 2A**). We observed a strong dose-dependent response in inhibiting OVA-specific CD8^+^ T cell proliferation in the dLNs (**Figure 2B**). In mice that received one dose, significantly fewer OTI cells were recovered from the challenge site dLNs of mice that received a mid (5 μg) or higher (20 μg) dose, but not a low (1 μg) dose. The same dose-dependent reduction in OTI was observed in mice that received two doses, with lowest OTI recovery in the mice that received the highest dose (2 x 20 μg), indicating that clonal deletion was more effective with a higher dose of antigen (**Figure 2B**). These results were consistent with OTI numbers recovered from the spleen, showing that even though T cell education takes place locally in the s.c. dLNs, a systemic tolerogenic response is generated (**Figure S2A**). Furthermore, it was necessary to increase the s.c. dose in order to attain the tolerogenic behavior observed with one dose of an i.v. injection (19), suggesting that a higher threshold to suppression exists in the LN and peripheral lymphatics compared to the liver and also that antigen dose is an important modulating factor (31, 32).

We then focused on the OTI and OTII cell phenotypes in experiments performed at the optimized dose of 20 μg s.c. and in the prime-boost regimen that generated the most effective OTI antigen non-responsiveness, and we assessed the tolerogenic responses induced by OVA-p(GluNAc) compared to unconjugated OVA. In this context, tolerance induction is characterized by an abrogated T-cell response to antigenic challenge and an enrichment of antigen-specific Treg cells. Five days post-challenge on day 22, s.c. prophylactic tolerization with OVA or OVA-p(GluNAc) both resulted in a significant reduction in OTI CD8^+^ T cell proliferation in the dLNs compared to untreated saline controls (**Figure 2C**). This result was comparable to that obtained in the spleen (**Figure S2B**). Even though OVA-p(GluNAc) did not lead to a significantly lower OTI recovery compared to unmodified OVA, it induced a number of tolerogenic signatures distinct from OVA-educated T cells. OTI cells primed with OVA-p(GluNAc) expressed significantly higher levels of co-inhibitory receptors, including PD-1 and Lag-3, compared with OVA (**Figures 2D, E** **)**. OTI cells from the OVA-p(GluNAc) group also highly expressed Tim-3, another co-inhibitory marker of exhaustion (**Figure 2F**). OVA-p(GluNAc) also induced a sizeable subset of OTI cells that co-express PD-1 and Tim-3 (**Figure S2C**), known to mark terminally exhausted cells in the context of tumors and chronic viral infections (33). Additionally, OTI cells from both the OVA and OVA-p(GluNAc) groups had significantly down-regulated the surface expression of their TCR, indicative of a self-inhibitory and anergic response (**Figure 2G**). Upon restimulation of OTI cells isolated from OVA-p(GluNAc)-treated mice with their cognate peptide OVA_257-264_ peptide *ex vivo*, a similar fraction produced the pro-inflammatory cytokine IFNγ (**Figure S2D**) but to a significantly lower extent illustrated by a 2-fold reduction in IFNγ MFI of the secretors (**Figure 2H**). OTI cells from the OVA-p(GluNAc) group secreted significantly higher levels of IL-10, an immunosuppressive cytokine known to play important roles in the induction and maintenance of tolerance (**Figure 2I**) (34).

We observed similar tolerogenic effects exerted in the OTII CD4^+^ T cell compartment. Upon antigenic challenge, fewer OTII cells were recovered from the dLNs in both the OVA and OVA-p(GluNAc) treated mice compared to untreated saline controls (**Figure 2J**). The OVA-p(GluNAc) treatment induced significantly higher CD4^+^ antigen-specific Foxp3^+^CD25^+^ Tregs (**Figure 2K**), as well as Foxp3^+^ST2^+^ Tregs (**Figure 2L**), both with major roles in tolerance (35). The OTII cells tolerized with OVA-p(GluNAc) more highly expressed co-inhibitory molecules such as Lag-3 (**Figure 2M**) and CTLA-4 (**Figure 2N**) and also down-regulated their TCR **(** **Figure 2O**). Next, we evaluated the effector function of the OTII cells upon *ex vivo* antigen reencounter, and detected cytokines either (1) produced by cells isolated from dLNs and spleen using flow cytometry after a 6-hour culture with their cognate OVA_323-339_ peptide, or (2) secreted into the culture supernatant using the LegendPlex assay after a 3-day culture with full OVA protein. LN cells from OVA-p(GluNAc)-treated mice had significantly reduced IL-13 production into the supernatant, suggesting that this treatment can also be useful in suppressing Th2-mediated reactions such as allergies (**Figure 2P**). There were also lower levels of Th17 cytokines, IL-17 and IL-22 secreted (**Figures S2E, F**). Furthermore, OTII cells from both the LN and spleen produced markedly lower levels of Th1 pro-inflammatory cytokines such as IFNγ, IL-2 and TNFα, indicating an ablation of their effector response (**Figure 2Q**). OVA-p(GluNAc) suppressed the presence of polyfunctional CD4^+^ T cells, measured by their ability to produce more than one cytokine, pointing to a dysfunctional state. The OTII cells also produced higher IL-10 levels (**Figure 2R**). Thus, we demonstrated that s.c. treatment with p(GluNAc) conjugated antigen generates antigen-specific tolerance, characterized by deletion, upregulation of surface co-inhibitory molecules, induction of both CD25^+^ (IL-2 receptor) and ST2^+^ (IL-33 receptor) Tregs, and an abrogation of broad-spectrum effector cytokines upon antigenic challenge.

### LN-Targeted Antigen-p(GluNAc) Conjugate Induces Tolerogenic Memory *via* CD8^+^ Regulatory Subsets That Can Suppress Adoptively Transferred Effector CD4+ T Cells

**Figure 3:**
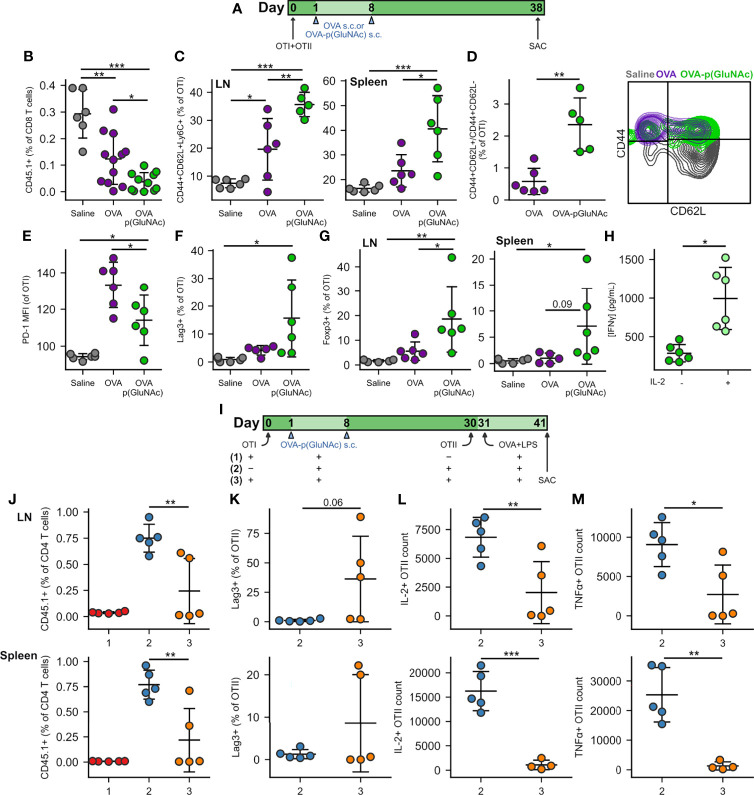
**(A)** CD45.2^+^ mice that had received an adoptive transfer of 1x10^6^ of both OTI (CD45.1^+^CD3^+^CD8^+^) and OTII (CD45.1^+^CD3^+^CD4^+^) T cells *via* i.v. injection, were treated s.c. in all four hocks on days 1 and 8 with saline, or 20 μg of OVA or OVA-p(GluNAc) (5 μg per hock). On day 38, all mice were sacrificed and the dLNs and spleen were analyzed for OTI and OTII T cell phenotype. **(B)** OTI CD8^+^ T cells recovered from dLNs. **(C)** Central memory OTI CD8^+^ T cells in dLNs (left) and spleen (right). **(D)** Ratio of central memory to effector memory OTI CD8^+^ T cells (left) and representative flow cytometry contour plot of the memory subsets (right) induced in the spleen. **(E)** PD-1 MFI on OTI CD8^+^ T cells in dLNs. **(F)** Lag-3^+^ OTI CD8^+^ T cells in dLNs. **(G)** Foxp3^+^ OTI CD8^+^ T cells in dLNs (left) and spleen (right). **(H)** Splenocytes from the OVA-p(GluNAc) group were restimulated with 100 μg/mL OVA in culture media alone or supplemented with 200 Units/mL (~12 ng/mL) exogenous IL-2, and IFNγ levels were measured in the supernatant 3 days later by ELISA. **(I)** CD45.2^+^ mice received a first adoptive transfer of 1x10^6^ OTI CD8^+^ T cells (groups 1,3) or no cells (group 2), followed by two s.c. OVA-p(GluNAc) treatments on days 1 and 8 for all groups. On day 30, mice from groups 2 and 3 received a second adoptive transfer of 5x10^5^ OTII CD4^+^ T cells. All mice were administered an OVA+LPS challenge on day 31, and 10 days later, the dLNs and spleen were examined for the OTII CD4^+^ T cell response. **(J)** OTII CD4^+^ T cells recovered from dLNs (top) and spleen (bottom). **(K)** Lag-3^+^ OTII CD4^+^ T cells in dLNs (top) and spleen (bottom). **(L)** Numbers of IL-2 producing OTII cells in dLNs (top) or spleen (bottom) after a 6-h *ex vivo* restimulation with OVA_323-339_ peptide. **(M)** Numbers of TNFα secreting OTII CD4^+^ T cells in dLNs (top) or spleen (bottom) after a 6-h *ex vivo* restimulation with OVA_323-339_ peptide. Data are pooled from two independent experiments (n= 5-12), and represent the mean ± SD. *p ≤ 0.05, **p ≤ 0.01, ***p ≤ 0.001 by one-way ANOVA using Tukey’s *post hoc* test in **(B, C, E–G, J)**, and unpaired Student’s T test in **(D, H, K–M)**.

We sought to further evaluate the mechanisms of action of LN-targeted OVA-p(GluNAc) by assessing suppressive populations induced in the long-term at steady-state (without an antigenic challenge), especially in the antigen-specific CD8^+^ T cell compartment. We treated mice s.c. with either unconjugated OVA or OVA-p(GluNAc), and evaluated the OTI phenotype in the dLNs and spleen one month following the booster injection (**Figure 3A**). At day 38, we observed a significantly lower recovery of OTI cells from the dLNs of OVA-p(GluNAc)-treated mice, indicating that the activated antigen-specific CD8^+^ T cells were deleted, resulting in a smaller pool of circulating cells (**Figure 3B**). We confirmed that OVA-p(GluNAc) treatment leads to a substantial initial proliferation of OTI cells, measured by CFSE dilution of circulating OTI in blood 3 days post-injection (**Figure S3A**), establishing that the deletion observed with s.c. OVA-p(GluNAc) was not due to incomplete priming by LN APCs but rather abortive proliferation, similar to the mechanism observed with liver-targeted OVA-p(GluNAc) (19). Thus, p(GluNAc) conjugation enhanced clonal deletion as its tolerogenic mechanism, a phenomenon observed not only in adoptively transferred T cells but also in endogenous autoimmune disease models (36).

We next investigated whether circulating antigen-specific regulatory memory was preferentially induced in CD8^+^ T cells educated by p(GluNAc) conjugated antigen, compared to free antigen, in the dLNs. Surviving OTI cells educated by OVA-p(GluNAc) exhibited a central memory phenotype characterized by high expression of CD44, CD62L and Ly6C in the dLNs (**Figure 3C**, left). OTI cells from the spleen also shared this phenotype, further validating that local antigen education in the dLNs is able to generate a circulating central memory T cell pool poised for immune suppression (**Figure 3C**, right) (37, 38). Not only did OVA-p(GluNAc) lead to more central memory CD8^+^ T cells overall but the proportion of central memory cells (CD44^+^CD62L^+^) compared with effector memory cells (CD44^+^CD62L^-^) was significantly higher (**Figure 3D**).

In contrast to what we observed after challenge, OTI cells in the OVA-p(GluNAc) group had a lower PD-1 expression at steady-state (**Figure 3E**), possibly because of the absence of chronic inflammatory stimuli and feedback networks that are usually needed to maintain high PD-1 expression and an exhausted state (39). Contrarily to PD-1, Lag-3 was expressed at high levels on OTI cells (**Figure 3F**), indicating that other mechanisms exist to maintain its expression even in the absence of residual antigen or chronic inflammation, which might be through interaction with scavenger receptor LSECtin (Clec4g) expressed on LECs (40).

Importantly, we noticed a significant induction in CD8^+^ T cells that were Foxp3^+^, both in the dLNs and spleen of mice that had been treated with OVA-p(GluNAc) (**Figure 3G**). Along with antigen-specific Foxp3^+^ CD25^+^ and Foxp3^+^ ST2^+^ CD4^+^ Tregs, these could also be the source of the heightened IL-10 levels secreted upon antigenic challenge (**Figure 2I**). Foxp3-expressing CD8^+^ Tregs have been reported to be important suppressive players in autoimmune disease such as type 1 diabetes and especially in the context of transplantation where donor cells continue to express MHCI for long time periods following the graft (41). The anergic T cells were rescued in their ability to produce IFNγ by the addition of exogenous IL-2 in the restimulation culture supernatant (**Figure 3H**) (42). Since this was an ELISA measurement, it was not possible to point out the identities of the T cells that were most responsible for this reversal in effector function, but it is most likely due to both CD4^+^ and CD8^+^ T cells. A similar restoration or increase in cytokine production was observed when cells from the saline and OVA groups were also restimulated in the presence of additional IL-2 (**Figure S3B**). Nonetheless, tolerance induced by s.c. antigen-p(GluNAc) is long-lasting, as evidenced by the resistance to antigenic challenge three months following the tolerization dose (**Figure S3C**).

We next asked whether the antigen-specific Foxp3^+^ CD8^+^ T cells induced by OVA-p(GluNAc) were capable of suppressing antigen-specific effector CD4^+^ T cells. We set up three groups to answer this question. Group #1 received a first adoptive transfer of OTI cells, followed by the OVA-p(GluNAc) tolerizing treatment and antigenic challenge but not the second OTII adoptive transfer (positive control for tolerance). Group #2 did not receive a first adoptive transfer of OTI cells but received the OVA-p(GluNAc) treatment, followed by a second adoptive transfer of OTII cells and challenge (negative control for tolerance). Experimental group #3 received both the first and second adoptive transfers, including OVA-p(GluNAc) treatments and the antigenic challenge. The purpose of the challenge following the second adoptive transfer was to activate the naïve CD4^+^ T cells into an effector phenotype. Moreover, we chose to wait an additional 10 days prior to sacrificing the mice to give the OTI CD8^+^ Tregs enough time to encounter the effector OTII cells in the face of a potentially overwhelming LPS-induced inflammatory environment (**Figure 3I**). We found that OTII cells from group #3 were significantly suppressed compared to OTII cells from group #2 at day 41. OTII cells from the dLNs and spleen of group #3 were recovered in smaller numbers (**Figure 3J**), more highly expressed Lag-3 (**Figure 3K**), and were impaired in their ability to produce IL-2 and TNFα cytokines upon restimulation with their cognate peptide (**Figures 3L, M** **)**. This shows that antigen-specific CD8^+^ Tregs induced with p(GluNAc)-conjugated antigen are long-lived and contribute to suppression of antigen-specific effector CD4^+^ T cells.

### Inhibition of LAG-3 Signaling Completely Reverses CD4^+^ and CD8^+^ T Cell Tolerance Induced by LN-Targeted OVA-p(GluNAc)

**Figure 4:**
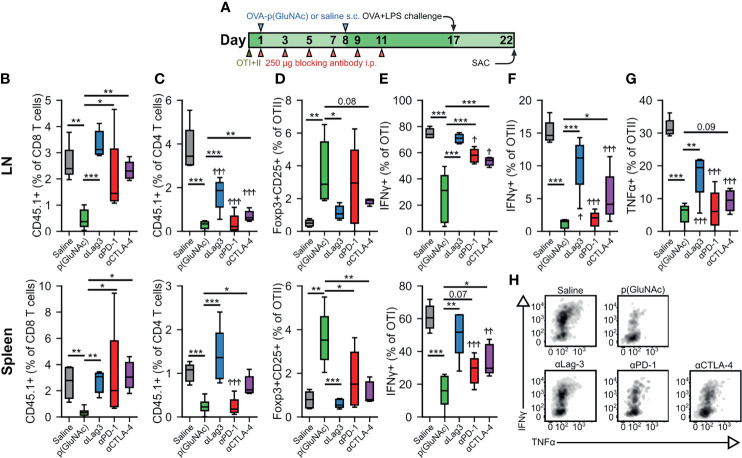
**(A–F)** CD45.2^+^ mice that had received an adoptive transfer of 1x10^6^ of both OTI (CD45.1^+^CD3^+^CD8^+^) and OTII (CD45.1^+^CD3^+^CD4^+^) T cells *via* i.v. injection, were treated on days 1 and 8 with saline, or 20 μg of OVA-p(GluNAc) s.c. in all four hocks (5 μg per hock). On days 1, 3, 5, 7, 9 and 11, mice were also treated with 250 μg of either αLag-3, αPD-1 or αCTLA-4. On day 17, mice were given a s.c. OVA+LPS challenge, and were sacrificed 5 days later to evaluate the OTI and OTII T cell phenotype in the dLNs and spleen. **(B)** OTI CD8^+^ T cells recovered from dLNs (top) and spleen (bottom). **(C)** OTII CD4^+^ T cells recovered from dLNs (top) and spleen (bottom). **(D)** Antigen-specific OTII CD4^+^ Tregs in dLNs (top) and spleen (bottom). **(E)** IFNγ secreting OTI CD8^+^ T cells after a 6-h *ex vivo* restimulation with OVA_257-264_ peptide from dLNs (top) and spleen (bottom). **(F)** IFNγ producing OTII CD4^+^ T cells after a 6-h *ex vivo* restimulation with OVA_323-339_ peptide. **(G)** TNFα secreting OTII CD4^+^ T cells after a 6-h *ex vivo* restimulation with OVA_323-339_ peptide. **(H)** Representative flow cytometry plots depicting IFNγ^+^ and TNFα^+^ OTI CD8^+^ T cells from dLNs after a 6-h *ex vivo* restimulation with OVA_257-264_ peptide. Data are pooled from two independent experiments (n= 5-10), and box-and-whisker plots represent the median, first and third quartiles. Statistical differences w.r.t saline were determined by one-way ANOVA using Dunnett’s *post hoc* test, and one-way ANOVA using Tukey’s *post hoc* test w.r.t OVA-p(GluNAc). Stars above horizontal bars represent p values with respect to the OVA-p(GluNAc) group (*p ≤ 0.05, **p ≤ 0.01, ***p ≤ 0.001) and † indicate p values with respect to the saline group (^†^p ≤ 0.05, ^† †^p ≤ 0.01, ^† † †^p ≤ 0.001).

We have demonstrated that OTI CD8^+^ and OTII CD4^+^ T cells engage the co-inhibitory module by up-regulating several surface immunosuppressive molecules, including PD-1 and Lag-3. We thus sought to investigate the role of these co-inhibitory signaling pathways in the tolerogenic mechanism of action of LN-targeted antigen-p(GluNAc) glycoconjugates. We set up a tolerance experiment as described above, but where we administered, during the OVA-p(GluNAc) priming window, i.p. injections of 250 μg blocking antibody against either Lag-3, PD-1 or CTLA-4, or no antibody for a total of 6 injections (**Figure 4A**). We challenged the mice with OVA and LPS 6 days following the last dose of blocking antibody and assessed the impact on the OTI and OTII T cell response 5 days after challenge. The antigen-specific CD8^+^ T cell deletional tolerance established with OVA-p(GluNAc) was completely ablated to the non-tolerized saline levels when Lag-3, PD-1 or CTLA-4 was blocked in both the dLNs and spleen, though a slightly larger effect was observed with Lag-3 neutralization (**Figure 4B**). Antigen non-responsiveness was also reversed in the OTII compartment but not to levels seen in the saline-treated mice, except with αLag-3 and αCTLA-4 in the spleen (**Figure 4C**). Antigen-specific CD4^+^ Treg induction was also abrogated and diminished back to saline levels, especially with Lag-3 neutralization (**Figure 4D**). Upon restimulation with OVA_257-264_ peptide, IFNγ production by OTI CD8^+^ T cells was completely restored with αLag-3 but only partially with αPD-1 and αCTLA-4 (**Figure 4E**). Similar trends were observed in IFNγ and TNFα production in the OTII CD4^+^ T cell compartment, albeit not to equivalent levels as with OTI (**Figures 4F–H**). OTII CD4^+^ T cell cytokine impairment was not rescued in the spleen, indicating that there is more of a CD4^+^ T cell local effect in the dLNs (**Figures S4A, B**). Therefore, these inhibitory signaling pathways investigated are important axes of T cell tolerance induced by LN-targeted antigen-p(GluNAc) glycoconjugates.

### OVA-p(GluNAc) Presentation to CD4^+^ T Cells and Cross-Presentation to CD8^+^ T Cells Is Mediated by Dendritic Cells

**Figure 5:**
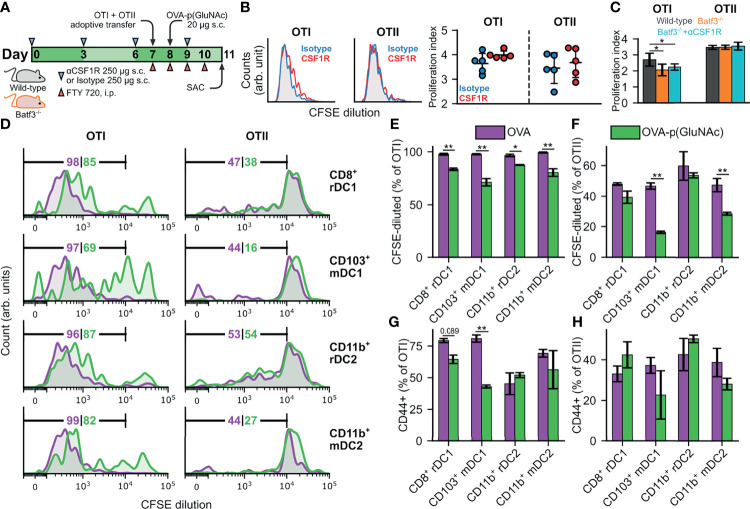
**(A)** CD45.2^+^ mice of wild-type (WT) or Batf3^-/-^ genotype were treated s.c. in all four hocks with 250 μg of αCSF1R or an isotype IgG2a control on days 0, 3, 6 and 9. On day 7, mice received an adoptive transfer of CFSE-labeled OTI CD8^+^ T and OTII CD4^+^ T cells *via* i.v. injection, followed by a s.c. administration of 20 μg OVA-p(GluNAc) on day 8, and daily i.p. injections of FTY 720 inhibitor starting on day 7. On day 11, mice were sacrificed and the dLNs and spleen were examined for OTI and OTII proliferation. **(B)** (Left) Representative flow cytometry histograms of the CFSE dilution undergone by OTI CD8^+^ T (left) and OTII CD4^+^ T (right) cells in the dLNs of WT mice in the isotype control (blue) and αCSF1R (red) conditions. (Right) Quantitative analysis of the OTI and OTII T cell proliferation index in dLNs of WT mice treated as described above. **(C)** Quantitative analysis of the OTI and OTII T cell proliferation index in dLNs of Batf3^-/-^ mice and WT mice treated as described above. **(D–H)** DCs were FACS sorted from s.c. LNs (axillary, brachial, inguinal, popliteal, cervical) of WT mice into four populations: CD8^+^ resident (CD8^+^ rDC1), CD103^+^ migratory (CD103^+^ mDC1), CD11b^+^ resident (CD11b^+^ rDC2) and CD11b^+^ migratory (CD11b^+^ mDC2), and stimulated *in vitro* in a 1:1 ratio with CFSE-labeled OTI CD8^+^ T and OTII CD4^+^ T cells in the presence of 2 μM of OVA or OVA-p(GluNAc). 3 days later, the OTI and OTII T cells were analyzed for proliferation and activation (CD44^+^). **(D)** Representative flow cytometry histograms of the CFSE dilution (numbers indicate percent proliferated) undergone by OTI CD8^+^ T (left) and OTII CD4^+^ T (right) cells induced by each DC subset in the OVA (purple) and OVA-p(GluNAc) (green) groups. **(E)** Quantitative analysis of the OTI CD8^+^ T proliferation. **(F)** Quantitative analysis of the OTII CD4^+^ T proliferation. **(G)** CD44^+^ OTI CD8^+^ T cells. **(H)** CD44^+^ OTII CD4^+^ T cells. The graphs show means ± SD, n = 5. *p ≤ 0.05, **p ≤ 0.01 by unpaired Student’s T test in B, one-way ANOVA using Tukey’s *post hoc* test in C, and two-way ANOVA using Sidak’s *post hoc* test in **(E–H)**.

The biodistribution experiment described in **Figure 1** showed that several professional and semi-professional APCs were responsible for antigen-p(GluNAc) uptake in the dLNs, but their contribution to antigen presentation to naïve CD4^+^ and CD8^+^ T cells in the dLNs remained to be elucidated. To tease out the contribution of specific APC subsets to antigen presentation, we evaluated the proliferation of OTI and OTII cells 3 days post-s.c. immunization in transgenic mice that lacked the APC subsets of interest or in wild type C57BL/6 mice where those APC subsets were depleted using monoclonal antibodies. We first focused our attention on macrophages, which we showed are major uptakers (**Figures 1F, G**). We compared the initial proliferative response of OTI and OTII cells in wild type mice that received 250 μg of anti-CFS1R depleting antibody or isotype control s.c. on days 0, 3, 6 and 9. These mice received an adoptive transfer of CSFE-labeled OTI and OTII cells on day 7 and 20 μg OVA-p(GluNAc) s.c. on day 8. They were also administered daily i.p. injections of FTY-720 inhibitor to trap the T cells in the LNs in order to maximize exposure of the T cells to peptide-bearing MHC expressing APCs (**Figure 5A**). A problem with antibody depletion such as with anti-CSF1R is the systemic dissemination associated with i.v. or i.p. injections of the antibody (43). In order to limit macrophage depletion to the dLNs, we administered the antibody s.c. in the hocks in the same way that we immunized the animals. We found that, compared with clodronate depletion, this local antibody injection depleted macrophage populations of interest, namely CD169^+^ SCS and medullary macrophages as well as more deeply located TZMs, in LNs only but left splenic macrophages intact (**Figure S5A**). We observed extensive but similar OTI and OTII proliferation in both the αCSF1R-treated and isotype-treated mice, indicating that LN macrophages are dispensable to the priming of CD4^+^ and CD8^+^ T cells in response to s.c. administered antigen-p(GluNAc) (**Figure 5B**).

To determine the contribution of another major uptaker, cross-presenting DCs, to s.c. OVA-p(GluNAc) immunization, we used Batf3^-/-^ mice that lack cross-presenting CD8^+^ DCs (44). We verified that there were minimal residual DCs in the LNs of these mice due to compensatory Batf1 expression (**Figure S5B**). We followed the same schedule as described above (**Figure 5A**). OTI cells proliferated significantly less in the Batf3^-/-^ mice compared to wild type mice, showing that these DCs play an important role in the cross-presentation of OVA-p(GluNAc); OTII cells were unaffected, as anticipated (**Figure 5C**). To confirm that macrophages were not involved, we further depleted these subsets through s.c. αCSF1R antibody injections in Batf3^-/-^ mice according to the above-described schedule (**Figure 5A**) and observed no further change in proliferation of OTI cells (**Figure 5C**). Even though we identified that cross-presenting DCs were important, they are evidently not the only APC involved, since we obtained non-negligible residual OTI proliferation in the Batf3^-/-^ mice (**Figure 5C**).

Because we still saw substantial OTI proliferation in Batf3^-/-^ mice, we sorted DC subsets from WT LNs and assessed their ability to present GluNAc-delivered OVA to T cells *in vitro* in order to identify the important DC players. We isolated the subcutaneous LNs (axillary, brachial, inguinal, popliteal, cervical) from wild-type mice and sorted the LN digests into four populations: CD8^+^ resident (CD11c^+^MHCII^int^CD8^+^CD11b^-^, denoted as CD8^+^ rDC1), CD103^+^ migratory (CD11c^+^MHCII^high^CD103^+^CD11b^-^, denoted as CD103^+^ mDC1), CD11b^+^ resident (CD11c^+^ MHCII^int^CD8^-^CD11b^+^, denoted as CD11b^+^ rDC2) and CD11b^+^ migratory (CD11c^+^MHCII^high^CD103^-^CD11b^+^, denoted as CD11b^+^ mDC2). We then stimulated each population *in vitro* in a 1:1 ratio with CFSE-labeled OTI and OTII cells in the presence of 2 μM of unmodified OVA or OVA-p(GluNAc). 3 days later, the OTI and OTII cells were analyzed for proliferation and activation (antigen experience), measured by dilution of the CFSE dye and CD44 expression, respectively. We made four main observations: (1) OVA-p(GluNAc) presentation elicited mainly a CD8^+^ T cell response (i.e. proliferation and activation), (2) presentation was not limited to cross-presenting DC1s, but DC2s were also important, (3) LN-resident subsets were more important than migratory populations for both DC1s and DC2s and, (4) OVA-p(GluNAc) generally resulted in a lower OTI and OTII proliferation and activation compared to unmodified OVA, indicative of an early tolerogenic skewing of T cell fate (**Figures 5D–H**). We also assessed the ability of LECs (the other major uptaker) to present OVA-p(GluNAc), and, while they did, they did so to a lower extent compared to DCs (**Figure S5C**). Thus, we established that DCs, alongside being good uptakers, are also the main LN APC involved in presenting s.c. administered antigen-p(GluNAc) to naïve CD4^+^ and CD8^+^ T cells.

## Discussion

Our previous work has demonstrated that synthetically glycosylated antigen may be useful as an inverse vaccine platform for inducing antigen-specific tolerance (19). The versatility and mild conditions of the antigen conjugation chemistry to our glycopolymer ensure that the strategy can be universally applied to any antigen that contains a native or engineered primary amine. A synthetically-glycosylated inverse vaccine has now entered phase I clinical trials for inducing tolerance in the context of celiac disease (ClinicalTrials.gov Identifier: NCT04248855). Although our previous work investigated targeting hepatic APCs (19), in this study we investigate tolerance induction mediated by targeting LN-resident APCs accessed through s.c. injection.

LNs are the site of tightly orchestrated responses that can be guided toward immunity or tolerance, depending on context (45). Similar to the liver, antigen dose and frequency, formulation and co-formulation with modulatory signals, as well as specific APC players determine the immunological response (46). Here, we focus on understanding the mechanisms of action of synthetically glycosylated inverse vaccines on LN-resident APCs, and to what extent dose and dose frequency may need to be adapted to achieve tolerance. Delivering the antigen conjugated to a glycopolymer may be beneficial for lymphatic absorption and channeling to LN-resident APCs and then for uptake *via* binding to their scavenger receptors to promote tolerance.

When delivered s.c., antigen conjugated to p(GluNAc), in this case OVA-p(GluNAc), rapidly drains and accumulates in the dLNs, to a significantly higher extent than unmodified OVA, which is consistent with particle filtration dynamics in the dLNs (**Figure 1E**). Glyco-polymerization alters the physicochemical properties of the antigen in important ways: the molecular weight is increased by 30-70 kDa, resulting in a net neutrally charged, branched polymeric particle. These nanoparticles drain into the lymphatics and accumulate in the dLNs, whereas smaller particles may be rapidly filtered through floor lymphatic endothelial cells and into systemic circulation *via* high endothelial venules, and larger microparticles may be preferentially captured by migratory APCs at the site of injection for subsequent trafficking to the dLNs (21, 47). However, given that OVA alone resulted in similar tolerogenic outcomes as OVA-p(GluNAc) in some instances, such as in total numbers of OTI and OTII recovered upon challenge, it is possible that OVA is taken up and processed by APCs more rapidly than OVA-p(GluNAc), resulting in negligible signal at the measured timepoints (**Figure 1E**). Thus, conjugation to p(GluNAc) may not significantly lengthen the residence time in the LNs but may only delay enzymatic cleavage in the endosome. This mechanistic distinction may be further explored by repeating the experiment described in **Figure 1E** using DQ-OVA instead of OVA.

Uptake of synthetically glycosylated antigen by APCs is mediated through the carbohydrate binding domain of various C type lectin and scavenger receptors and can be inhibited by the addition of free sugars in media (19). We analyzed the immgen database (http://www.immgen.org/) for the expression of several scavenger and lectin receptors involved in the uptake of carbohydrates, including GluNAc-terminated residues, among APCs targeted by OVA-p(GluNAc), and found that they were broadly expressed, but to different extents on these cell types (**Figure S1B**). We identified Asgr1 and 2 to be only minor players in LN APCs compared to hepatic APCs (48). Clec4g (LSECtin) was found to be highly expressed exclusively on LECs, justifying their high uptake of OVA-p(GluNAc) and their similarity in scavenging profile to liver sinusoidal endothelial cells (49). Other receptors found highly expressed by the hematopoietic APCs were DEC-205 (Ly75), which has been explored as an antigen target for the induction of tolerance (50) and Clec9a, primarily found as apoptotic scavenger receptor by cross-presenting DCs (51). LECs and macrophages share MARCO expression, which has been used for antigen targeting in tolerance induction (17, 52). The mannose receptor (Mrc1), which can promiscuously bind GluNAc glycosylated antigen, was also highly expressed on LECs (53). This analysis also revealed shared receptors between LECs and macrophages, which reflects their synergy in scavenging in the LN subcapsular sinus, similar to the parallels between sinusoidal endothelial cells and Kupffer cells in the liver (54). Thus, by virtue of size, retention, expression of C-type lectin and scavenger receptors, LN APCs are able to effectively take up synthetically glycosylated antigen.

Compared to the liver or oral mucosa, where immune responses are skewed toward tolerance because of the abundance of oral or gut antigen that need to be interpreted in an innocuous manner, immune responses to exogenous antigens in the peripheral lymphatics usually aim to generate an inflammatory response in the context of an infection. However, LNs under homeostasis do continually drain self-antigen from the local tissue, and this constant antigen exposure may be important in maintaining peripheral tolerance. For example, in mice lacking skin-draining lymphatics, skin-specific autoimmunity was observed to develop (55). Furthermore, LECs have an essential role in the maintenance of tolerance to peripheral tissue-transcribed antigens *via* the deletion of autoreactive cells or the generation of autoantigen-specific CD4^+^ Tregs, thereby acting as an additional mechanism to compensate for potentially autoreactive T cells that escape central tolerance (56–58). LECs can also induce tolerance to exogenous antigens draining from peripheral sites of immunization, inflammation and tumors, through direct antigen presentation to both naïve CD8^+^ and CD4^+^ T cells (59, 60). This tolerogenic antigen presentation is accompanied by the up-regulation of co-inhibitory molecules, as well as soluble mediators such as IDO that can directly suppress T cells and prevent APCs from maturing and presenting antigen to produce effectors (61).

Canonical Foxp3^+^CD25^+^ Tregs play a crucial role in ensuring the maintenance of tolerance and, more recently, antigen-specific Tregs induced in the periphery are being increasingly recognized as important regulators (62, 63). We have also shown the dependence of LN-targeted suppression on long-lived CD8^+^ regulatory T cell subsets (**Figures 3I–M**). These constitute an important arm in the natural control of autoimmunity (64) but can also be induced under different treatment conditions that have mostly been investigated in immune-privileged sites (65) and in the context of transplantation and peptide immunotherapy in lupus (66). The ability of antigen-p(GluNAc) to result in broad antigen-specific regulatory and suppressor subsets of T cells would be a highly desirable property.

Memory has been found to contribute beneficially or harmfully to the maintenance of tolerance in a context-dependent manner. In type 1 diabetes, lower avidity auto-reactive clones have been shown to adopt a central memory phenotype that serves to regulate antigen presentation and activation of destructive high-avidity autoreactive clones in the pancreatic dLNs (38). Memory CD8^+^ T cells have also been shown to promote tolerance to graft through nitric oxide production (37). We see a similar phenomenon at play where antigen-specific CD8^+^ T cells that survived deletion post-antigen-p(GluNAc)-mediated abortive proliferation preferentially differentiate into a central memory state (**Figures 3C, D**) where they can mediate suppression to future antigenic challenge (**Figures 2**, **S3C**). In future mechanistic studies, it will be of interest to evaluate the contribution of TCF1^+^ stem-cell like memory to the central memory compartment and tolerogenic state induced by s.c. antigen-p(GluNAc) administration (67).

There were noticeable differences in the response of antigen-specific CD4^+^ and CD8^+^ T cells to blockade of the distinct co-inhibitory pathways (Lag-3, PD-1 and CTLA-4). CD8^+^ T cell tolerance was significantly more ablated when these signaling pathways were disrupted, indicating a higher dependence on these signaling pathways for tolerance induction (**Figure 4**). All three pathways were found to be important to some extent for CD4^+^ and CD8^+^ T cell tolerance, a result that did not surprise us given that many of these co-inhibitory molecules form part of an immunosuppressive module co-regulated by overlapping signaling such as IL-27 (33). Lag-3 was found to be an essential suppressive pathway responsible for inducing deletional tolerance in CD8^+^ T cells in both the dLNs and spleen and in CD4^+^ T cells in the spleen (68). This also suggested to us that other signaling axes exist to ensure CD4^+^ T cell peripheral tolerance is maintained. One example is considering how Lag-3 expressed on CD4^+^ T cells interacts with its ligands in the LN microenvironment. We have shown that OVA-p(GluNAc)-educated OTII cells express higher Lag-3 levels (**Figure 2M**). Lag-3 binds to MHCII on various APCs, an interaction that contributes to CD4^+^ T cell activation and is not blocked by the αLag-3 (C9B7W) antibody that we used in our experiments (69). Lag-3 on T cells has also been reported to interact with LSECtin that is highly expressed on LN-LECs (**Figure S1B**) (40).

This is the first report of local LN macrophage depletion using a s.c. injection of CSF-1R depleting antibody, but s.c. administration is a recently validated strategy for the locoregional enrichment of blocking antibodies such as checkpoint antibodies in the sentinel LNs for tumor control (70). Francis et al. demonstrated that s.c. administration of αPD-1 or αCTLA-4 antibodies ipsilateral to the primary tumor results in accumulation in the local dLNs and anti-tumor efficacy but also a systemic abscopal effect. While we observed a robust decrease in macrophage subsets in the dLNs, we did not suppress macrophages in the spleen, indicating that antibodies rapidly drain to and are retained in the dLNs where they exert a local effect, leading to a systemic immunological response. While our data showed a dispensable role for macrophages in glycoconjugate-medicated antigen priming, it is possible that macrophages relay the acquired antigen to DCs for further processing and presentation onto MHC, as has been reported (71). This coordinated effort and transfer of antigen between different APC subsets through vesicular routes has been evidenced under steady-state (72) and, more recently, elegantly demonstrated in the context of sentinel LN priming in cancer (73).

Dendritic cells have unique and varied intrinsic pathways of antigen presentation but can also be highly cooperative, depending on context (74). For example, mannose receptor-directed antigen is channeled to early endosomes and the cross-presentation pathway (75). Even though the current paradigm is that DC1s (LN-resident CD8^+^ or migratory CD103^+^) are specialized in cross-presenting antigen to CD8^+^ T cells, while DC2s (LN-resident or migratory CD11b^+^) are better equipped to present to CD4^+^ T cells, all DCs are capable of presenting to both CD4^+^ and CD8^+^ T cells given the right circumstances dictated by location (both anatomically and within the LN), antigen dose and administration route, and inflammatory stimulus (24, 76–79). Consistent with this, we found that *ex vivo* priming with OVA-p(GluNAc) by DC1s and DC2s resulted in both OTI and OTII expansion and activation but primarily a CD8^+^ T cell response with at least a two-fold difference in OTI proliferation, compared with OTII (**Figures 5D–F**).

The divergence in CD4^+^ and CD8^+^ T cell proliferation is not surprising given that they have very different activation requirements (80). For instance, CD4^+^ T cell proliferation is more dependent on prolonged antigen exposure compared to CD8^+^ T cells (81). The APC antigen uptake and distribution landscape is also instrumental to regulating differential priming (82). Furthermore, while CD4^+^ T cells are required for optimal CD8^+^ T cell activation during a primary activation or memory recall response and for survival (83), CD8^+^ T cell memory formation has been shown to be intrinsic and CD4^+^ T cell independent (84). In the context of peripheral tolerance, CD4^+^ T cell help is usually an instigator of autoreactive CD8^+^ T cell effector function in several autoimmune conditions such as in type 1 diabetes and is undesirable in transplant tolerance (85–87). Since the antigen-specific CD4^+^ and CD8^+^ T cells were both in contact with the sorted DCs at the same time in our *ex vivo* sorting and priming experiment, the CD4^+^ T cell help provided by OTII cells could be an additional factor that contributed to the OTI proliferation (**Figures 5D, E**). The OTI and OTII proliferation was elicited by both sorted DC1 and DC2 populations, especially LN-resident subsets, which is what we expected given that OVA-p(GluNAc) drains rapidly to the LN and is not retained at the s.c. site of injection 72 h post-injection, which is the timeframe for when migratory DCs make their way to dLNs with captured antigen (**Figure S1A**).

In conclusion, in this work, we present a novel approach of inducing antigen-specific tolerance using synthetically glycosylated antigen *via* peripheral s.c. routes of targeting. We leverage the biophysical, biochemical and immunological environment of the LN and its cellular players to induce robust and lasting prophylactic tolerance to an exogenous antigen. This strategy has powerful implications in the prophylaxis and treatment of autoimmune and inflammatory diseases.

## Data Availability Statement

The raw data supporting the conclusions of this article will be made available by the authors, without undue reservation.

## Ethics Statement

The animal study was reviewed and approved by Institutional Animal Care and Use Committee at the University of Chicago.

## Author Contributions

JH and MS oversaw all research. CM, EW, MS, and JH designed the research strategy. DW conceptualized materials. DW and MR synthesized materials. CM, SC, EW, AS, MN, JR, and HN-S performed experiments. CM analyzed experiments. CM and JH wrote the manuscript. All authors contributed to the article and approved the submitted version.

## Funding

This work was supported in part by seed funding from the Chicago Immunoengineering Innovation Center at the University of Chicago as well as the NIH (1R01CA219304 to MS).

## Conflict of Interest

DW and JH are inventors of patents related to synthetically glycosylated inverse vaccines, licensed to Anokion, Inc., in which DW, MS, and JH are shareholders and for which JH consults and is a member of the Board of Directors, and to Lanta Bio SA, in which MS and JH are shareholders.

The remaining authors declare that the research was conducted in the absence of any commercial or financial relationships that could be construed as a potential conflict of interest.

## Publisher’s Note

All claims expressed in this article are solely those of the authors and do not necessarily represent those of their affiliated organizations, or those of the publisher, the editors and the reviewers. Any product that may be evaluated in this article, or claim that may be made by its manufacturer, is not guaranteed or endorsed by the publisher.
