# Supplementary material for: Lymph Node-Targeted Synthetically Glycosylated Antigen Leads to Antigen-Specific Immunological Tolerance
Source: Front Immunol. 2021 Sep 24;12:714842. doi: 10.3389/fimmu.2021.714842 (PMC8498032; doi:10.3389/fimmu.2021.714842)
Supplement: Supplementary file 1 [file DataSheet_1.docx]

**Title**: Lymph node-targeted synthetically glycosylated antigen leads to antigen-specific immunological tolerance

**Authors**: Chitavi D. Maulloo^1^, Shijie Cao^1^, Elyse A. Watkins^1^, Michal M. Raczy^1^, Ani. S. Solanki^2^, Mindy Nguyen^1^, Joseph W. Reda^1^, Ha-Na Shim^1^, D. Scott Wilson*^1,4^, Melody A. Swartz*^1,3,5,6^ and Jeffrey A. Hubbell*^1,3,6^

**Affiliations**:

^1^Pritzker School of Molecular Engineering, University of Chicago, Chicago, IL 60637

^2^Animal Resources Center, University of Chicago, Chicago, IL 60637

^3^Committee on Immunology, University of Chicago, Chicago, IL 60637

^4^Biomedical Engineering Department, Johns Hopkins University, Baltimore MD 21205

^5^Ben May Department of Cancer Research, University of Chicago, Chicago, IL 60637

^6^Committee on Cancer Biology, University of Chicago, Chicago, IL 60637

*Correspondence to: scott.wilson@jhmi.edu, melodyswartz@uchicago.edu, jhubbell@uchicago.edu

**Supplemental Data:**

Figure S1. Characterization of lymphatic drainage from the s.c. injection site, LN APC subsets and expression of GluNAc-binding receptor candidates on these subsets.

Figure S2. Antigen-p(GluNAc) leads to antigen-specific CD4^+^ and CD8^+^ T cell tolerance in the spleen in addition to dLNs, and suppresses Th17 responses.

Figure S3. LN-targeted antigen-p(GluNAc) induces extensive initial proliferation of CD8^+^ T cells and subsequent long-lived tolerogenic memory.

Figure S4. Antigen-specific CD4^+^ T cell effector function is conserved in the spleen of mice treated with s.c. antigen-p(GluNAc) and antibodies blocking LAG-3, PD-1 and CTLA-4.

Figure S5. Macrophage subsets are effectively depleted in the dLNs of mice injected s.c. with αCSF1R, and are not responsible for antigen-p(GluNAc) priming to naïve T cells.

Figure S6*.* Flow cytometry gating strategy for OTI and OTII T cells.

Figure S7*.* Flow cytometry gating strategy for regulatory, inhibitory, exhaustion, and anergy receptors on OTII CD4^+^ T cells.

Figure S8*.* Flow cytometry gating strategy for cytokine producing OTII CD4^+^ T cells after a 6-h ex vivo restimulation with OVA_323-339_ peptide.

Figure S9*.* Flow cytometry gating strategy for regulatory, inhibitory, exhaustion, and anergy receptors on OTI CD8^+^ T cells, and cytokine producing OTI CD8^+^ T cells after a 6-h ex vivo restimulation with OVA_257-264_ peptide.


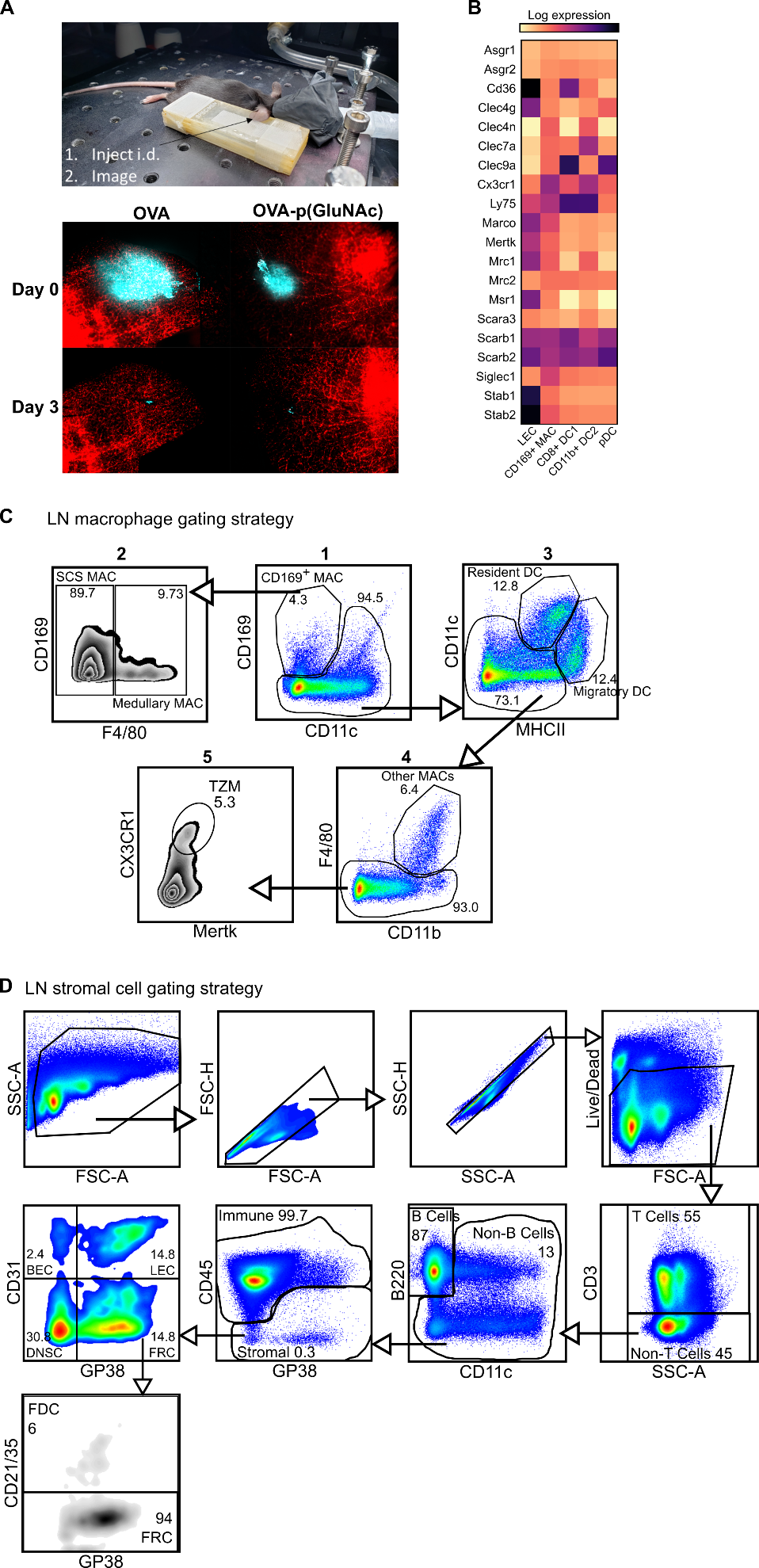


**Figure S1. *Characterization of lymphatic drainage from the s.c. injection site, LN APC subsets and expression of GluNAc-binding receptor candidates on these subsets.*** (A) ProxTom (Prox1-tdTomato) mice were injected in the ear dermis with OVA_647_ or OVA_647_-p(GluNAc). The ear dermis around the site of injection was imaged on the day of injection (day 0) and 3 days post-injection using *in vivo* fluorescence microscopy. Lymphatics are shown in red, and OVA antigen is displayed in cyan. (B) Heatmap showing the comparative expression of different classes of carbohydrate-binding and scavenger receptors on LECs, CD169^+^ macrophages, CD8^+^ DC1s, CD11b^+^ DC2s and plasmacytoid DCs in the s.c. LNs of wild-type male C57BL/6 mice. Data analyzed from the immgen database (http://www.immgen.org/). (C) Representative flow cytometry plots showing the gating strategy used for LN macrophage subsets. (D) Representative flow cytometry plots showing the gating strategy used for LN stromal cell subsets.


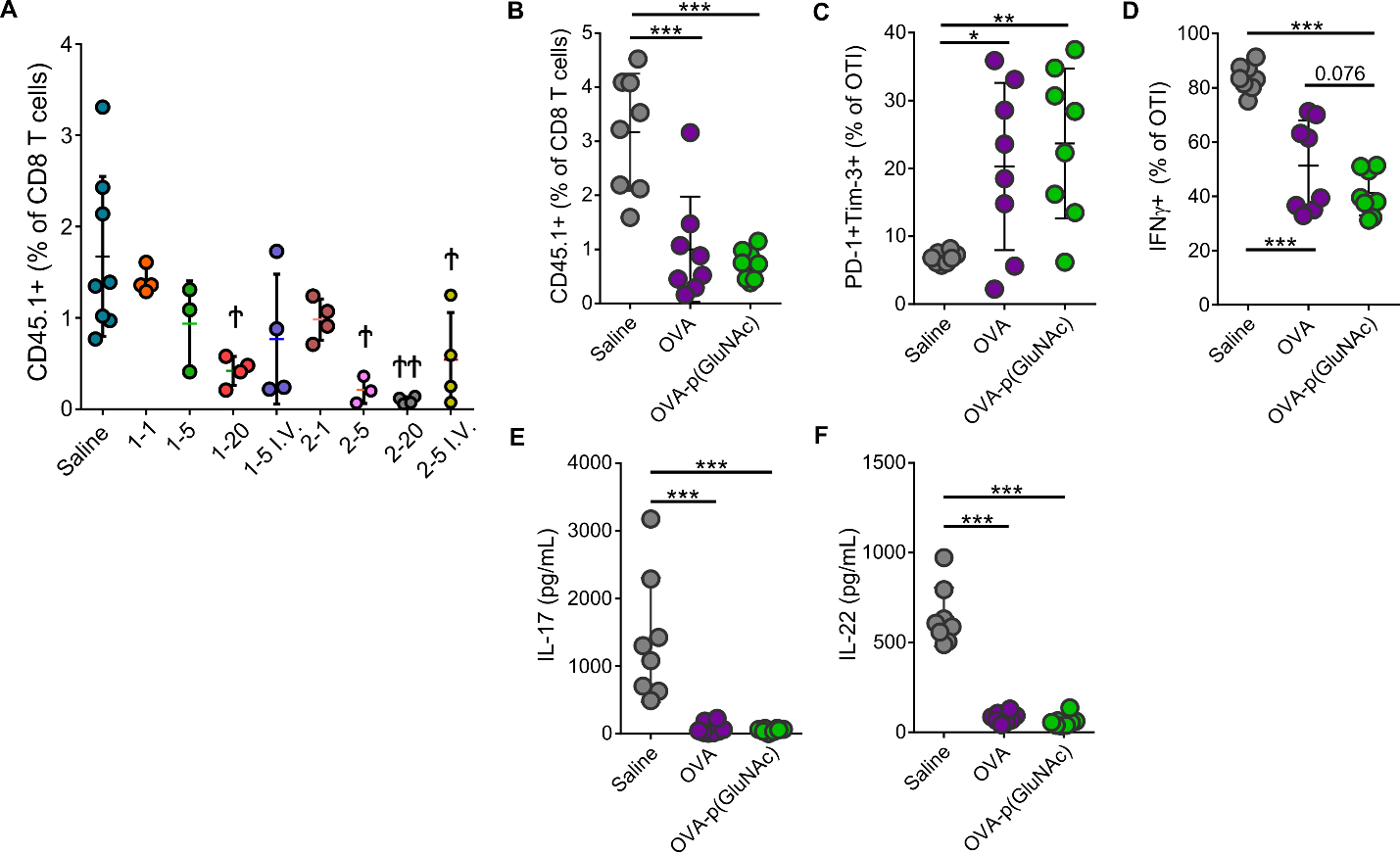


**Figure S2. *Antigen-p(GluNAc) leads to antigen-specific CD4^+^ and CD8^+^ T cell tolerance in the spleen in addition to dLNs, and suppresses Th17 responses.*** (A) Experimental set-up described in Figure 2A. % OTI cells recovered from the spleen at time of sacrifice. Plot legends are as follows: 1-1 (1 μg s.c., once), 15 (5 μg s.c., once), 1-20 (20 μg s.c., once), 1-5 i.v. (5 μg i.v., once), 2-1 (1 μg s.c., twice), 2-5 (5 μg s.c., twice), 2-20 (20 μg s.c., twice) and 2-5 i.v. (5 μg i.v., twice). (B) % OTI cells recovered from the spleen at day 22. (C) % PD-1^+^Tim-3^+^ (terminally exhausted) OTI cells in the spleen at day 22. (D) % IFNγ secreting OTI cells after a 6-h ex vivo restimulation with OVA_257-264_ peptide. (E) IL-17 levels in the supernatant of LN cells restimulated with 100 μg/mL OVA protein for 4 days, measured by LegendPlex^TM^ assay. (F) IL-22 levels in the supernatant of LN cells restimulated with 100 μg/mL OVA protein for 4 days, measured by LegendPlex^TM^ assay. Data represent mean ± SD. Statistical differences were determined by one-way ANOVA using Tukey’s post hoc test (*p ≤ 0.05, **p ≤ 0.01, ***p ≤ 0.001).


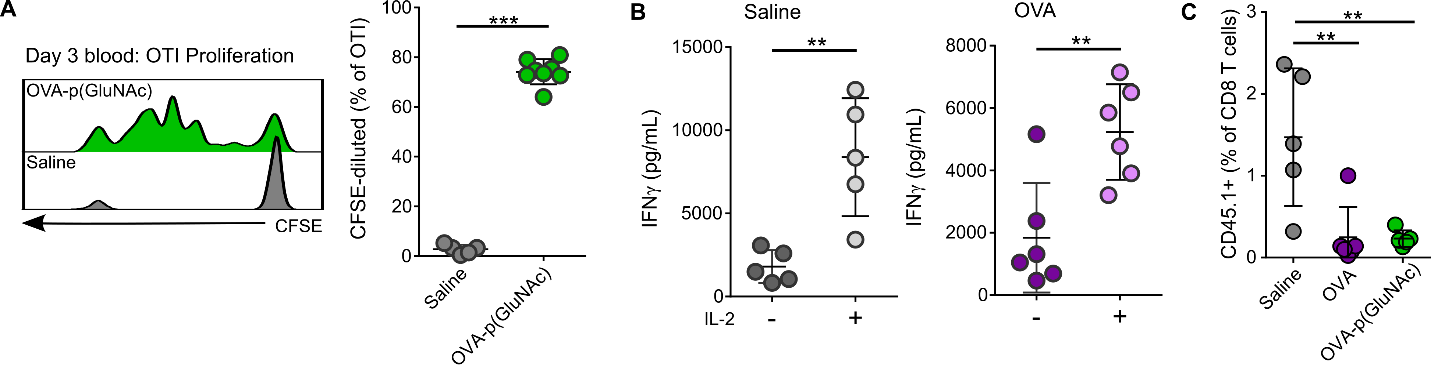


**Figure S3*. LN-targeted antigen-p(GluNAc) induces extensive initial proliferation of CD8^+^ T cells and subsequent long-lived tolerance.*** (A) (Left) Representative flow cytometry histograms of the CFSE dilution (proliferation) undergone by OTI cells in the blood of wild-type mice 3 days post-s.c. injection with saline or OVA-p(GluNAc). (Right) Quantitative analysis of the OTI proliferation. (B) Experimental set-up described in Figure 3A. Splenocytes from the saline or OVA group were restimulated with 100 μg/mL OVA in culture media alone or supplemented with 200 Units/mL (~12 ng/mL) exogenous IL-2, and levels of IFNγ were measured in the supernatant 3 days later by ELISA. (C) Mice were immunized as described in Figure 3A, received an OVA+LPS challenge 3 months following the second dose, and were sacrificed 5 days post-challenge. % OTI cells recovered from dLNs. Statistical differences were determined by unpaired Student’s T test in A, B, and one-way ANOVA using Tukey’s post hoc test in C (*p ≤ 0.05, **p ≤ 0.01, ***p ≤ 0.001).


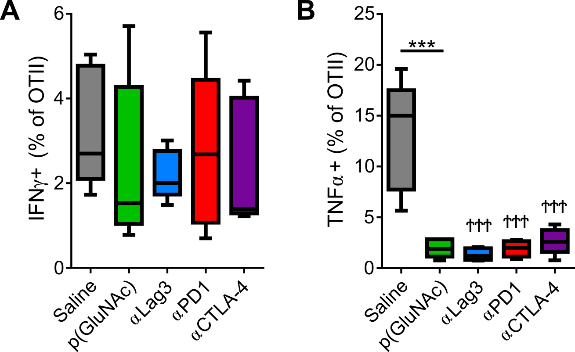


**Figure S4*. Antigen-specific CD4^+^ T cell effector function is conserved in the spleen of mice treated with s.c. antigen-p(GluNAc) and antibodies blocking LAG-3, PD-1 and CTLA-4.*** (A) Experimental set-up described in Figure 4A. % IFNγ producing splenic OTII cells after a 6-h ex vivo restimulation with OVA_323-339_ peptide. (B) % TNFα secreting splenic OTII cells after a 6-h ex vivo restimulation with OVA_323-339_ peptide. Box-and-whisker plots represent the median, first and third quartiles. Statistical differences were determined by one-way ANOVA using Dunnett’s post hoc test. Stars above horizontal bars represent p values with respect to the OVA-p(GluNAc) group (*p ≤ 0.05, **p ≤ 0.01, ***p ≤ 0.001) and † indicate p values with respect to the saline group († p ≤ 0.05, †† p ≤ 0.01, ††† p ≤ 0.001).


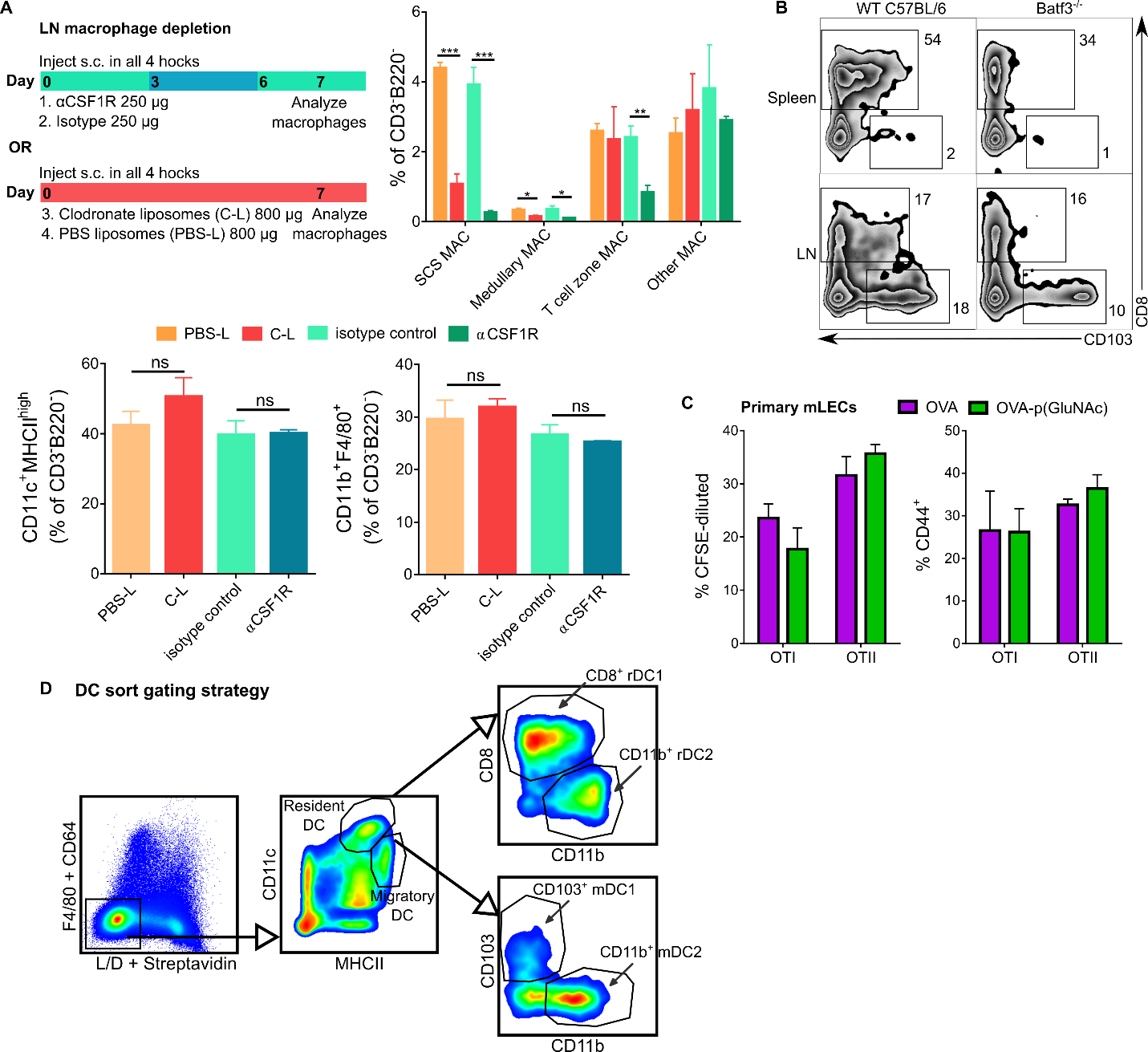


**Figure S5*. Macrophage subsets are effectively depleted in the dLNs of mice injected s.c. with αCSF1R, and are not responsible for antigen-p(GluNAc) priming to naïve T cells.*** (A) (Top left) Wild-type mice were treated s.c. in all four hocks with 250 μg of αCSF1R or an isotype IgG2a control on days 0 and 3, or alternatively, with 800 μg of clodronate or PBS-loaded liposomes on day 0. On day 7, mice were sacrificed and the dLNs and spleen were examined for the presence of macrophage subsets and DCs. (Top right) % macrophages in each subset in the dLNs. (Bottom left) % DCs in the dLNs. (Bottom right) % splenic macrophages. (B) Representative flow cytometry plots showing the reduction, but not complete absence, of CD8^+^ rDC1 and CD103^+^ mDC1 in the s.c. LN and spleen of Batf3^-/-^ mice. Populations shown are gated on CD11c^+^MHCII^high^. (C) LN-LECs were isolated from wild-type mice and expanded in vitro before they were stimulated in a 1:1 ratio with CFSE-labeled OTI and OTII cells in the presence of 2 μM of unmodified OVA or OVA-p(GluNAc). 3 days later, the OTI and OTII cells were analyzed for proliferation and activation (CD44^+^). Quantitative analysis of the OTI and OTII proliferation (left) and activation quantified by % CD44^+^ (right) in the conditions described above. (D) Representative flow cytometry plots showing the gating strategy used for sorting LNs into the four DC populations described in Figure 5E-I. The graphs show means ± SD. Statistical differences were determined by two-way ANOVA using Tukey’s post hoc test in A (top right) and one-way ANOVA using Tukey’s post hoc test in A (bottom) and C (*p ≤ 0.05, **p ≤ 0.01, ***p ≤ 0.001).


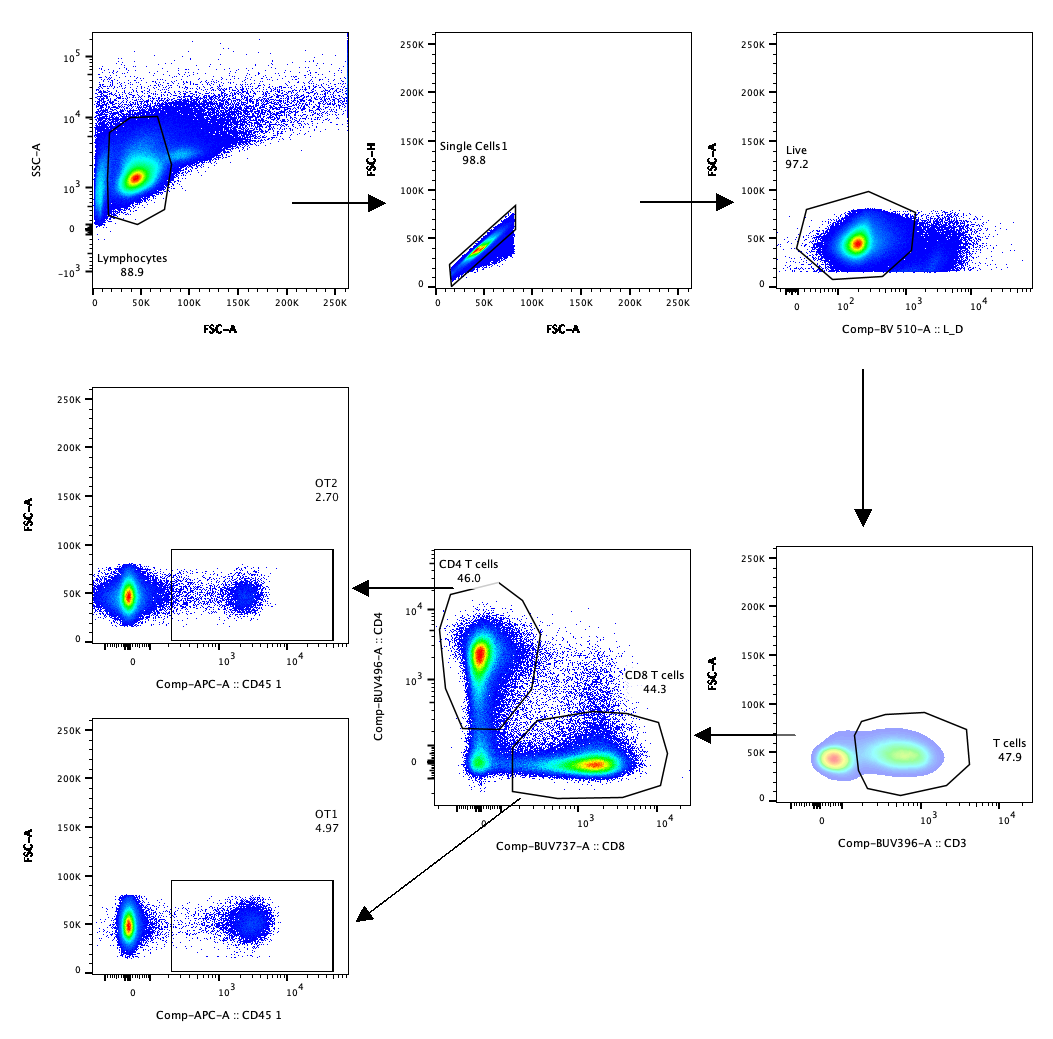


**Figure S6*.* Flow cytometry gating strategy for OTI and OTII T cells.**

**
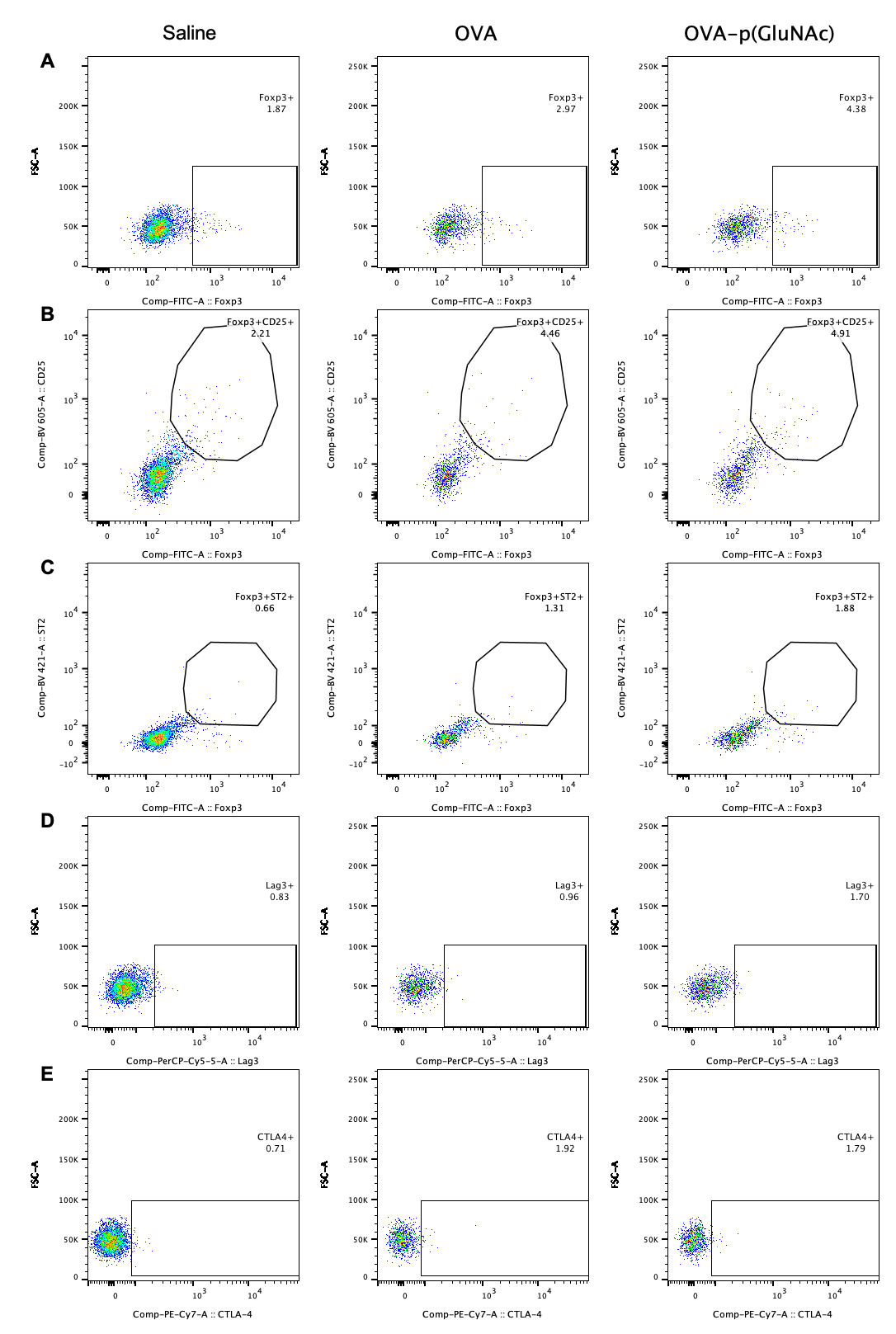
**

**Figure S7*.* Flow cytometry gating strategy for regulatory, inhibitory, exhaustion, and anergy receptors on OTII CD4^+^ T cells.** Gating strategy on OTII CD4^+^ T cells from saline, OVA, or OVA-p(GluNAc) treated group for **A)** FoxP3^+^ regulatory T cells, **B)** FoxP3^+^CD25^+^ regulatory T cells, **C)** FoxP3^+^ST2^+^ regulatory T cells, **D)** Lag-3^+^, **E)** CTLA-4^+^.

**
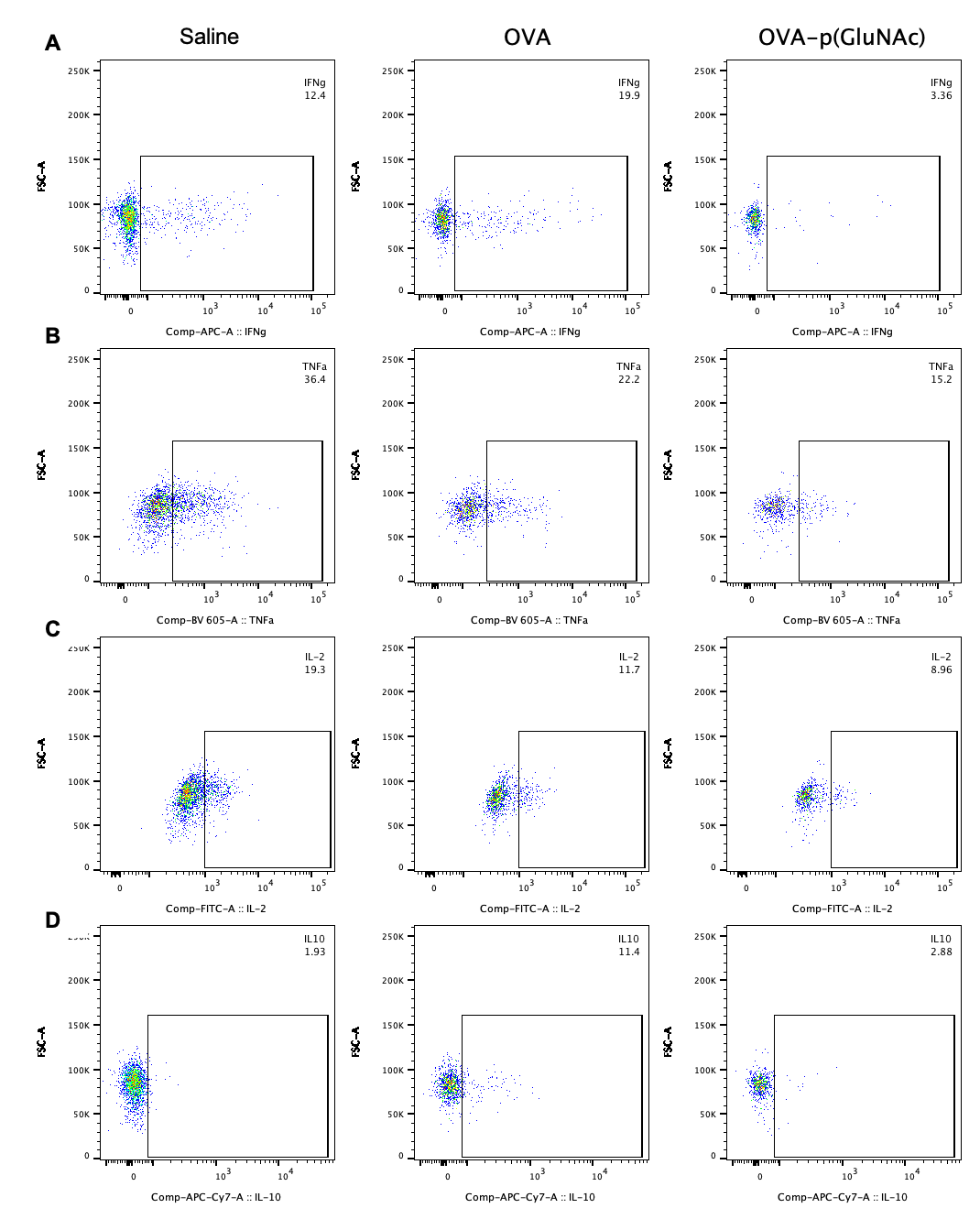
**

**Figure S8*.* Flow cytometry gating strategy for cytokine producing OTII CD4^+^ T cells after a 6-h ex vivo restimulation with OVA_323-339_ peptide.** Gating strategy on OTII CD4^+^ T cells from saline, OVA, or OVA-p(GluNAc) treated group producing **A)** IFNγ, **B)** TNFα, **C)** IL-2, and **D)** IL-10.

**
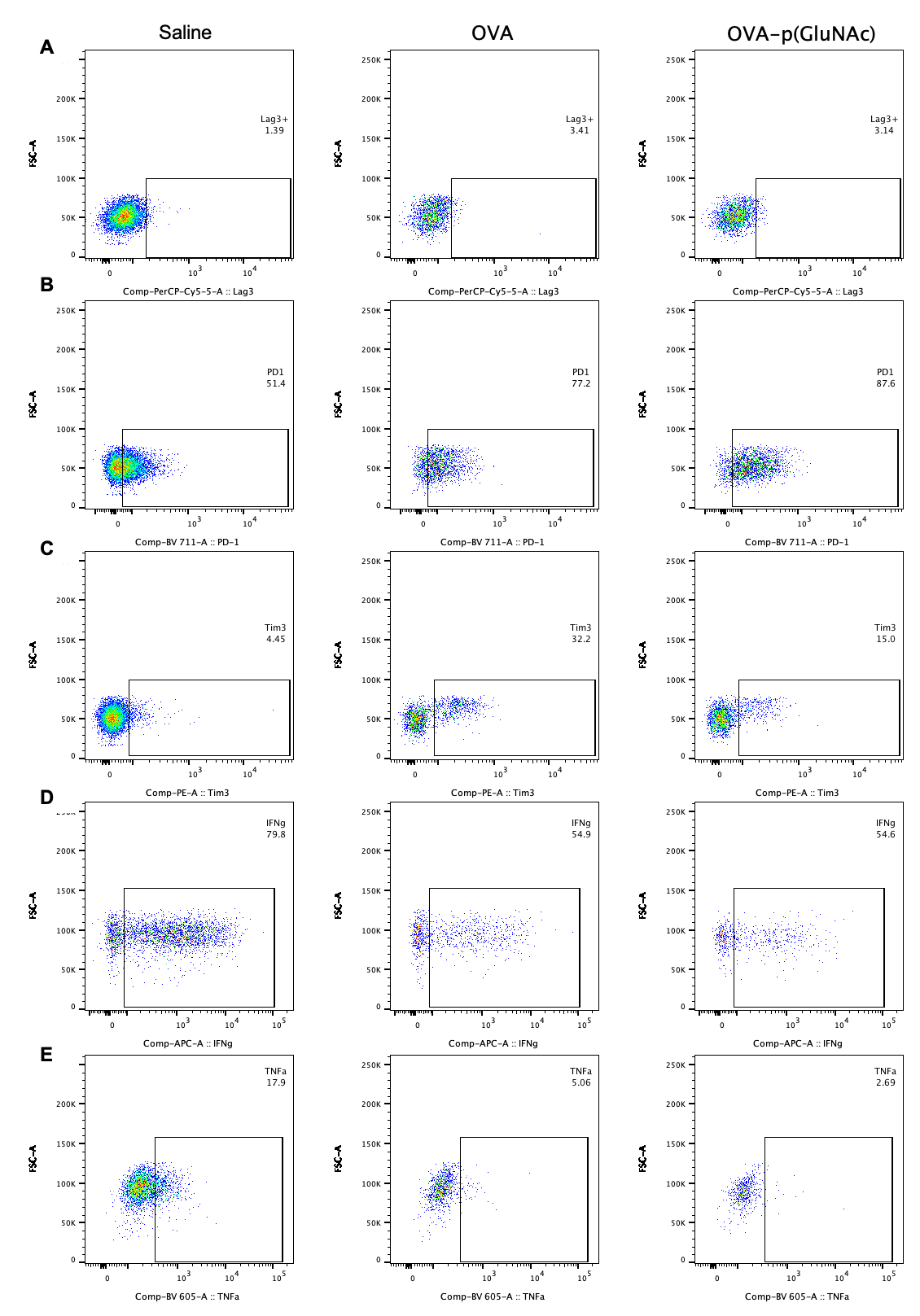
**

**Figure S9*.* Flow cytometry gating strategy for regulatory, inhibitory, exhaustion, and anergy receptors on OTI CD8^+^ T cells, and cytokine producing OTI CD8^+^ T cells after a 6-h ex vivo restimulation with OVA_257-264_ peptide.** Flow cytometry gating strategy on OTI CD8^+^ T cells from saline, OVA, or OVA-p(GluNAc) treated group for **A)** Lag-3^+^, **B)** PD-1^+^, and **C)** Tim3^+^. Gating strategy on **D)** IFNγ, and **E)** TNFα producing OTI CD8^+^ T cells.
